# Targeting epigenetic regulators induces transcription-replication conflicts to overcome ATR inhibitor resistance

**DOI:** 10.1093/nar/gkag811

**Published:** 2026-08-20

**Authors:** Samah W Awwad, Holly Barber, Josie Coulthard, Simon Lam, Alejandra Rojas, Nadia Gueorguieva, Michael Woods, Gabriel Balmus, Rimma Belotserkovskaya, Stephen P Jackson

**Affiliations:** Cancer Research UK Cambridge Institute, University of Cambridge, Cambridge CB2 0RE, United Kingdom; The Gurdon Institute, Cambridge CB2 1QN, United Kingdom; Cancer Research UK Cambridge Institute, University of Cambridge, Cambridge CB2 0RE, United Kingdom; Department of Biochemistry, University of Cambridge, Cambridge CB2 1QW, United Kingdom; Cancer Research UK Cambridge Institute, University of Cambridge, Cambridge CB2 0RE, United Kingdom; Cancer Research UK Cambridge Institute, University of Cambridge, Cambridge CB2 0RE, United Kingdom; The Gurdon Institute, Cambridge CB2 1QN, United Kingdom; Cancer Research UK Cambridge Institute, University of Cambridge, Cambridge CB2 0RE, United Kingdom; Cancer Research UK Cambridge Institute, University of Cambridge, Cambridge CB2 0RE, United Kingdom; The Gurdon Institute, Cambridge CB2 1QN, United Kingdom; Cancer Research UK Cambridge Institute, University of Cambridge, Cambridge CB2 0RE, United Kingdom; The Gurdon Institute, Cambridge CB2 1QN, United Kingdom; UK Dementia Research Institute, Cambridge CB2 0AH, United Kingdom; UK Dementia Research Institute, Cambridge CB2 0AH, United Kingdom; Department of Clinical Neurosciences, University of Cambridge, Cambridge CB2 0QQ, United Kingdom; Department of Molecular Neuroscience, Transylvanian Institute of Neuroscience, Cluj-Napoca 400157,Romania; Cancer Research UK Cambridge Institute, University of Cambridge, Cambridge CB2 0RE, United Kingdom; The Gurdon Institute, Cambridge CB2 1QN, United Kingdom; Cancer Research UK Cambridge Institute, University of Cambridge, Cambridge CB2 0RE, United Kingdom; The Gurdon Institute, Cambridge CB2 1QN, United Kingdom; Department of Biochemistry, University of Cambridge, Cambridge CB2 1QW, United Kingdom

## Abstract

Many cancer cells exhibit elevated replication stress and are highly dependent on ataxia telangiectasia and Rad3-related (ATR) kinase activity to maintain genomic stability and survival. While ATR inhibitors (ATRi) have great promise as therapeutic agents, intrinsic or acquired resistance will likely be a significant challenge. We previously showed that loss of the RNA polymerase II Mediator subunits CDK8 and Cyclin C (CCNC) confers resistance to ATRi by suppressing transcription-dependent replication stress. To identify vulnerabilities that could be exploited to restore ATRi sensitivity in these resistant settings, we performed genome-wide CRISPR screens in wild-type and *CDK8*-deficient cells. These screens revealed impairment of epigenetic components HDAC3 or the PRC2 complex as synthetic vulnerabilities that enhance ATRi sensitivity, particularly in contexts of loss of CDK8 or CCNC. This ATRi sensitivity is driven by the induction of transcriptional dysregulation, leading to increased transcription–replication collisions, replication stress, and apoptosis upon ATR inhibition. Importantly, we show that HDAC3 loss limits growth of ATRi-resistant tumours *in vivo*. Moreover, pharmacological inhibition of HDAC3 or PRC2 phenocopies their genetic loss. Collectively, our findings highlight the therapeutic potential of targeting epigenetic regulators to induce transcriptional dysregulation and ensuing replication stress to overcome ATRi resistance.

## Graphical Abstract

**Graphical Abstract:**
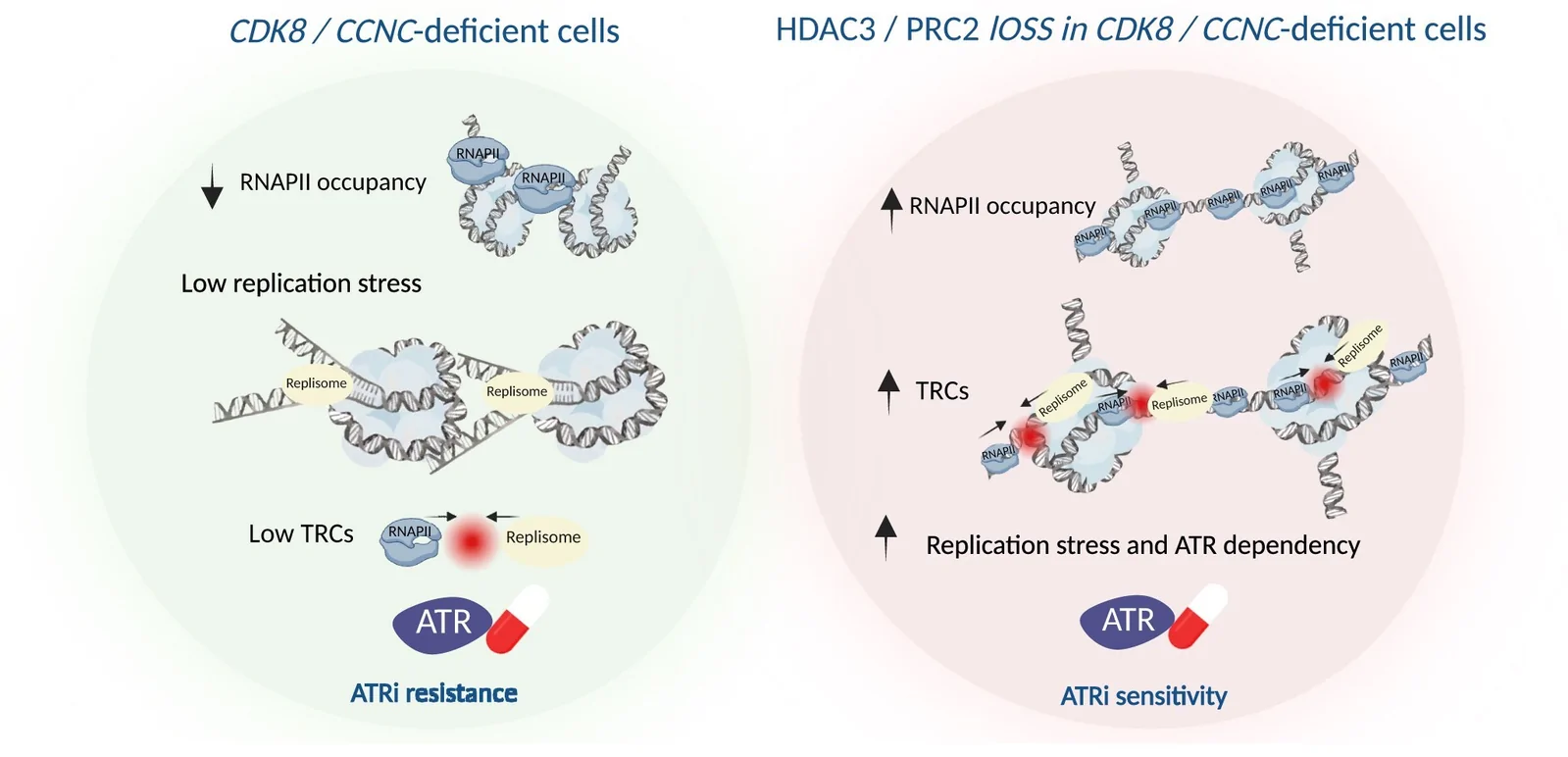

## Introduction

Cancer poses a major threat to human life and health. With the total number of cancer cases in the U.S. projected to rise by 50% by 2050 [1], and similar trends expected globally, more effective cancer therapies are urgently needed. Compounding this challenge, over 90% of cancer-related deaths are linked to resistance against chemotherapeutic drugs [2]. Therefore, overcoming drug resistance will be key to reducing mortality and managing the growing disease burden.

Replication stress (RS), defined as the slowing or stalling of replication fork progression, is a prevalent hallmark of cancer that is associated with DNA damage and genomic instability [3]. Various mechanisms underpin this stress [4], such as overexpression of oncogenes that result in excessive replication-origin firing and subsequent accumulation of replication-associated single-strand DNA gaps [5]. Furthermore, oncogene-dependent increases in transcription have been linked to transcription–replication conflicts and formation of DNA:RNA hybrids (R-loops) that pose barriers to the DNA replication machinery and can ultimately result in replication fork collapse and DNA double-stranded break (DSB) formation [6, 7]. Accordingly, cancer cells are often particularly reliant on the replication stress response (RSR) to maintain genomic integrity and promote survival.

The protein kinase ATR (ataxia telangiectasia and Rad3-related) and its primary target, CHK1 (Checkpoint kinase 1), are key regulators of the RSR. This pathway is further fine-tuned by the two related kinases WEE1 and PKMYT1, which act in concert with ATR/CHK1 [8, 9]. Together, these kinases safeguard genome integrity by slowing or arresting cell cycle progression until replication stress is resolved [8]. To capitalize on the dependence of tumour cells on ATR, multiple ATR inhibitors (ATRi) have been developed and are under clinical investigation [10–13]. ATRi are being explored predominantly as therapies for subsets of cancers that either experience high replication stress or have lost the related kinase, ATM [14–19]. Despite their promising clinical activities, it is not fully understood which pathways and genes influence ATRi efficacy and how clinical resistance mechanisms may arise. A deeper understanding of the signalling networks that modulate ATRi efficacy is therefore critical for maximizing therapeutic scope.

In this study, through genome-wide CRISPR knockout (KO) screens in wild-type (WT) and the ATRi-resistant *CDK8* KO human HAP1 cell lines, we discovered that either loss of histone deacetylase 3 (HDAC3) or disruption of the Polycomb repressive complex 2 (PRC2) complex through loss of key components such as EZH2, EED, and SUZ12, or loss of the PRC2-associated protein MTF2, selectively restores ATRi sensitivity. We show that codepletion of HDAC3 and CDK8 or CCNC induces transcription-associated replication stress and DNA damage, as evidenced by increased RNA polymerase (RNAP) II loading on chromatin and transcription–replication conflicts. Furthermore, our results show that inhibition of HDAC3 or the catalytic subunit of PRC2, EZH2, phenocopies their genetic loss in overcoming resistance to ATRi caused by *CDK8*/*CCNC* deficiency. Collectively, our data provide a potential strategy to circumvent ATRi resistance through combining ATRi with inhibitors of key epigenetic transcription regulators.

## Materials and methods

### Cell lines

All cell lines used in this study were routinely tested and confirmed to be free of mycoplasma contamination. Cells were cultured at 37°C in a humidified atmosphere with 5% CO_2_. Unless otherwise specified, all media were supplemented with 10% fetal bovine serum (FBS) and 1% penicillin–streptomycin (Supplementary Table S1).

To generate *CDK8/HDAC3* dKO HAP1 cells, *CDK8* KO #A17 HAP1 cells [20] were infected with LentiGuide-puro-NLS-GFP (DD5134; Addgene #185473) containing HDAC3 sgRNA2. Single clones were grown and confirmed by western blot and Sanger sequencing.

*CCNC* KO HAP1 cells were generated from HAP1 Cas9 expressing cells by infecting with LentiGuide-puro-NLS-GFP containing a signle guide RNA (sgRNA) targeting *CCNC*. Single cell-derived clones were selected, expanded, and confirmed by western blot and Sanger sequencing. This cell line was then used to generate *CCNC/HDAC3* dKO cell lines as described earlier.

*ARID1A* KO, *ATM* KO, *ARID1A/CDK8* dKO, *ARID1A/CCNC* dKO, and *ATM/CCNC* dKO HAP1 cells were generated from HAP1 Cas9 expressing cells by infecting with LentiGuide-puro-NLS-GFP containing the corresponsing sgRNA. sgRNAs used in this study are listed in Supplementary Table S2.

Flag-HDAC3 WT/Y298F-expressing HAP1 cells were established by transducing *CDK8/HDAC3* dKO and *CCNC/HDAC3* dKO HAP1 cells with HDAC3 WT or Y298F-3xFlag (synthesized by VectorBuilder). Cells were selected with 500 μg/ml G418, and expression was validated by western blotting.

RNaseH1-WT-expressing HAP1 (WT, *CDK8/HDAC3* dKO, and *CCNC/HDAC3* cells) cells were generated by lentiviral transduction using pLV-Tet-RNaseH1-WT-BFP (kind gift from Jacob Corn’s lab). Following infection, cells were maintained in tetracycline-negative FBS. RNaseH1 expression was induced by 10 ng/ml Doxycycline for 48 h. Induction and expression of RNaseH1 were confirmed by flow cytometry and western blot.

### Lentiviral generation and transduction

Lentiviral particles were produced by co-transfecting Lenti-X 293T cells with the plasmid of interest, psPAX2 (Addgene #12260), and pMD2.G (Addgene #12259) using TransIT-LT1 transfection reagent (MIR 2304). Virus-containing supernatant was collected 72 h post-transfection, filtered through 0.45 μm filter, and stored at −80°C. Target cells were transduced with the lentiviral supernatant in the presence of 8 μg/ml polybrene. Medium was changed 24 h post-transduction, and cells were selected with appropriate antibiotics as needed.

### Compounds

ATRi (AZD6738 and VE-821) and HDAC3 inhibitor (RGFP966) were obtained from SelleckChem. EZH2i (GSK126), EZH2i (EPZ-6438), and EED PROTAC (UNC6852) were obtained from Tocris.

### Genome-wide CRISPR-Cas9 screens

WT or *CDK8* KO HAP1-Cas9-expressing cells were transduced at a multiplicity of infection of 0.3 and 500-fold library coverage of the Brunello human genome wide sgRNA library. Twenty-four hours post-transduction, cells were selected with 1 μg/ml puromycin for 6 days to enrich for sgRNA expressing populations. Following selection, cells were split into two treatment arms; DMSO treated or treated with ATRi (AZD6738) at an IC20 for 10 population doublings. Cell pellets were collected at day 0 of treatment and at day 10 of treatment, genomic DNA was isolated using the QIAamp Blood Maxi Kit (Qiagen), and genome-integrated sgRNA sequences were amplified by polymerase chain reaction (PCR) using the Q5 Mastermix (New England Biolabs Ultra II) and i7 multiplexing barcoded primers. Products were sequenced on Illumina NovaSeq 6000 systems. Brunello sgRNA sequences were verified for off-target effects using Exorcise [21]. Guide enrichment analysis was performed using DrugZ [22] to compare DMSO-treated to ATRi-treated samples.

### Western blotting

Cells were lysed in Laemmli buffer [2% sodium dodecyl sulphate (SDS), 10% glycerol, 60 mM Tris–HCl, pH 6.8] and incubated for 10 min at 95°C followed by needle shearing of DNA. Proteins were resolved by SDS–polyacrylamide gel electrophoresis using 4%–12% Bis-Tris gels. Proteins were transferred onto Nitrocellulose membranes (Amersham) and blocked with 5% milk in Tris Buffered Saline – Tween20 (TBS-T), followed by incubation with primary antibodies (Supplementary Table S3) diluted in bovine serum albumin in TBS-T, overnight at 4°C. The membranes were washed with TBS-T and incubated with secondary antibodies in 5% milk for 1 h at room temperature. Following washing with TBS-T, membranes were developed using a ChemiDoc imaging system (Bio-Rad).

### Two-colour competitive cell growth assay

Cas9-expressing WT or *CDK8* KO HAP1 cells were transduced with a vector expressing sgRNA targeting HDAC3 (#1 or #3) coupled with GFP, or a control vector expressing RFP-sgLacZ. Following puromycin selection of transduced cells, equal quantities of GFP-sgHDAC3 and of RFP-sgLacZ expressing cells were mixed. Mixed populations were grown in the absence or presence of ATRi (AZD6738), and the proportions of GFP- and RFP-expressing cells were determined by flow cytometry at various timepoints over two weeks. CRISPR-editing efficiency after sgRNA transduction was tested by Sanger sequencing and analysed by TIDE.

### *In vitro* cell survival assays

For growth assays, HAP1 cells were plated as technical quadruplicates at either 2500 or 5000 cells/well in a 96-well plate. For drug survival assays, HAP1 cells were seeded at a density of 2500–5000 cells/well, U2OS and LNCaP cells were seeded at a density of 2000 cells/well, and C42 cells were seeded at a density of 1000 cells/well in 96-well plates. Sixteen to twenty-four hours later, drugs were added for 72 h for HAP1, 7 days for U2OS and C42, and 10 days for LNCaP cells. After the desired time had elapsed, Alamar Blue (Resazurin, Thermo Scientific 418900250) was added (20 μg/μl) and incubated at 37°C for 3 h. Analysis of dye conversion was measured using a CLARIOstar at an excitation/emission of 554/588 nm. The fluorescent signal is proportional to the number of living cells.

### Clonogenic cell survival assay

DLD1 cells were seeded at a density of 1000 cell per well in six-well plates in technical replicates. Twenty-four hours later, cells were treated with ATRi only, or a combination of ATRi and HDAC3i at the indicated doses. After 8 days, cells were fixed in 0.25% (wt/vol) crystal violet with 25% ethanol and washed with distilled water. Plates were scanned and colonies counted using FIJI based on colony area as previously described [23].

### Cell cycle analysis and flow cytometry

Cells were seeded at a density of 500k cells/well in six-well plates 16 h prior to treating. Cells were incubated with either DMSO or ATRi (AZD6738) for 24 h and, where indicated, pulse labelled with 10 mM EdU (5-ethynyl-2-deoxyuridine; Sigma–Aldrich 900 584) for 30 min prior to fixation. Cells were fixed permeabilized for 15 min with BD Cytofix/Cytoperm (BD bioscience) at room temperature. Antibody staining was performed in BD perm/wash buffer (BD biosciences) using the antibodies listed in Supplementary Table S3. The click reaction was subsequently performed before staining with 1 μg/ml DAPI in phosphate buffered saline (PBS) supplemented with 250 μg/ml RNase A. All samples were acquired using A5 FACS Symphony (BD Biosciences) and analysed with FlowJo v.10.10.0.

### DNA–RNA immunoprecipitation (DRIP-seq)

DNA–RNA immunoprecipitation (DRIP) assays were performed as previously described [24], in three biological replicates with the following amendments (during analysis we used two replicates of *CDK8/HDAC3* dKO cells in both DMSO and ATRi treated samples, as we omitted a low-quality replicate for each). HAP1 cells were seeded on 10-cm plates for 16 h followed by a 6 h treatment with 1 μM AZD6738. Cells were washed once with ice-cold PBS, lysed in cytoplasmic lysis buffer for 10 min on ice followed by a single wash with cytoplasmic lysis buffer. Nuclei were lysed in nuclei lysis buffer, and the lysate incubated for 1 h at 55°C with 300 rpm agitation. To precipitate the DNA, 3 M sodium acetate pH 5.2 was added (300 mM final) followed by transfer into 2.5× volume of 100% ethanol. The DNA was then washed in 85% ethanol, air-dried, and re-suspended in 1× TE buffer. Fifty micrograms of DNA was sonicated using a Diagenode Bioruptor on high settings for 13 cycles of 10 s on/30 s off, and sonication was confirmed by running the samples on a 1.5% agarose gel. All samples were equilibrated into 1× DRIP buffer, and 20 μg was immunoprecipitated overnight at 4°C with 5 μg S9.6 antibody pre-conjugated to 100 μl Dynabeads protein G. The beads were washed once in 1× DRIP buffer, once in 1× DRIP with 500 mM NaCl, once in LiCl buffer, and twice in 1× TE. DNA was eluted by incubation with 1× TE pre-added to 0.5% SDS and 0.5 mg/ml proteinase K for 30 min at 55°C. Eluted DNA was purified using phenol:chloroform:isoamyl alcohol, and following this, precipitated by ethanol. DNA was resuspended in 65 μl nuclease-free water. Five nanograms of DRIP DNA from each of three biological replicates from each condition were used for library preparation using the Thruplex DNA-seq kit for Illumina (TAKARA, R400674) according to the manufacturer’s protocol, using 12 PCR cycles for IP samples and 8 PCR cycles for input samples. Libraries were then sequenced as 2 lanes of S1 PE50 on the Illumina NovaSeq6000.

### DRIP-seq analysis

DRIP-seq reads were aligned to GRCh38 using HISAT2 [25] in paired-end mode and counted into bins of width 20 nt using samtools and igvtools and expressed as counts per million (CPM) total reads. Samples were taken in biological triplicate except for the *CDK8/HDAC3* dKO cells, both ATRi treated and untreated, which were taken in biological duplicate due to a quality issue affecting one replicate each.

For the metagene analysis, CPMs within −2 kb of the transcription start site (TSS) and +2 kb of the transcription end site (TES) of genes with a promoter-proximal peak according to ChIP-seq data (see RNAPII ChIP-seq analysis) were considered, excluding ribosomal RNA genes. Background was determined per sample by calculating the mean CPM in the region from −2 kb to the TSS and from the TES to +2 kb. CPMs were normalized by subtracting the background from the CPM. Fold enrichment was calculated per sample by dividing normalized CPMs by the background.

For the differential R-loop utilization analysis, mean CPM along gene bodies was calculated per gene. Two-tailed unpaired Wilcoxon rank-sum tests were performed on *CDK8* KO versus *CDK8/HDAC3* dKO samples. R version 4.2.3 was used in all analyses.

### ChIP-seq

ChIP was performed in biological quadruplicates. Ten million HAP1 cells were plated in 15-cm dishes 24 h prior to harvesting. Cells were crosslinked with 1% formaldehyde for 10 min at room temperature and quenched with 0.125 M Glycine for 5 min. After two washes with ice-cold PBS, cells were scraped and collected. Cell pellets were resuspended in 1 ml cytoplasmic buffer (50 mM HEPES, pH 7.5, 140 mM NaCl, 1 mM ethylenediaminetetraacetic acid (EDTA), 10% glycerol, 0.5% NP-40, 0.25% Triton) and incubated on ice for 10 min. Nuclei were pelleted by centrifugation at 1500 × *g* for 5 min, washed once with the same buffer, and lysed with nuclei lysis buffer (50 mM Tris, pH 8.0, 5 mM EDTA, 1% SDS) and incubated for 10 min on ice. Chromatin was sheared using a Bioruptor Plus sonicator (Diagenode Cat. No. B01020001) at high power for 16 cycles (30 s on, 30 s off). DNA fragment size (300–400 bp) was confirmed on a 2% agarose gel. Sonicated chromatin was cleared by centrifugation at 20 000 × *g* for 10 min at 4°C and diluted 1:10 with ChIP equilibration buffer (16.7 mM Tris, pH 8.0, 1.67 mM EDTA, 167 mM NaCl, 1.1% Triton). Chromatin was pre-cleared with Protein G Dynabeads (Invitrogen) for 1 h at 4°C with rotation. For immunoprecipitation, 3 μg of RNAPII or H3K27me3 antibodies were bound to 30 μl of Protein G Dynabeads in PBS at 4°C for 2 h with rotation. Beads were washed twice with PBS, resuspended in RIPA buffer (20 mM Tris, pH 8.0, 2 mM EDTA, 0.1% SDS, 1% Triton, 150 mM NaCl) and incubated with pre-cleared chromatin overnight at 4°C with rotation. Beads were washed twice with 1 ml RIPA buffer, then once with 1 ml high salt RIPA buffer (500 mM NaCl), 1 ml LiCl buffer (10 mM Tris, pH 8.0, 250 mM LiCl, 1 mM EDTA, 1% NP40), and finally twice with 1 ml TE buffer (10 mM Tris, pH 8.0, 1 mM EDTA, pH 8.0). Beads were resuspended with 100 μl nuclear lysis buffer, and 10% input samples were diluted to 1% final SDS concentration. All samples were incubated at 70°C overnight to reverse crosslink. Samples were then treated with 5 μl Proteinase K (20 mg/ml) at 60°C for 30 min, followed by RNaseA treatment (0.2 mg/ml) for 2 h at 37°C. DNA was purified twice with phenol-chloroform-isoamyl alcohol extraction, and precipitated with ethanol and sodium acetate. Purified DNA was used for library preparation with the ThruPLEX DNA-seq Kit (Takara, Cat. No. R400674) according to the manufacturer’s instructions. Libraries were sequenced on two lanes of 1.5B PE50 using an illumina NovaseqX sequencer.

### ChIP-seq analysis

Paired-end sequencing reads were aligned to human genome assembly GRCh38.p14 using HISAT2 [25] software, and read depth was calculated in 20 nt bins using samtools [26] and igvtools.

For the calculation of RNAPII occupancy and H3K27me3 histone marks along transcripts, ChIP-seq read density was counted from 2 kb upstream of TSS to 2 kb downstream of transcription termination sites (TTS). Read density for each sample was normalized by total read count. Density profiles for all transcripts were scaled between TSS and TTS and stacked to produce a metagene for each sample. Transcription start and termination sites were obtained from Ensembl [27] release 116.

To determine RNAPII processivity of transcripts, travelling ratios (TRs) of transcripts were calculated. This was defined as the density in the region spanning −30 to +300 bp about each TSS divided by the density from +300 bp from the TSS to the TTS. We calculated ratios of TRs of each transcript as the TR in a KO sample divided by the TR in the WT. R version 4.5.3 and Bioconductor GenomicRanges version 1.50.0 were used to calculate and visualize metagenes, TRs, and ratios of travelling ratios.

### Determination of head-on and co-directional transcription–replisome collisions

Origins of replication and leading strand direction were determined as previously described [28]. Briefly, previously published [29] OK-seq reads were obtained from the Gene Expression Omnibus with accession numbers SRR7109016 and SRR7109017. Sequences were trimmed using Trim Galore! [30] version 0.6.11 and aligned to the human genome assembly GRCh38 Ensembl release 116 using HISAT2. Duplicate reads were removed using SAMtools version 1.23 with options fixmate -m and then markdup -r. Replication fork directions were determined using OKseqHMM [31] version 2.0.0 with read coverage threshold 6 for SRR7109016 and 1 for SRR7109017. Peaks present in both datasets used to identify replication initiation zones using HOMER [32] version 4.8.2 with option mergePeaks -d given identifying origins of replication as the centres of initiation zones. Distances between origins and the nearest TSS were calculated using annotatePeaks.pl from HOMER and TSS coordinates from GRCh38 Ensembl release 116. R version 3.6.3 was used to run OKseqHMM. R version 4.5.3 was used for all other processing.

TSSs were oriented in sense direction and stacked in rank order of distance to the closest origin from most positive indicating co-directional (CD) travel of RNA polymerases and replisomes to most negative indicating head-on (HO) travel. RNAPII ChIP-seq peaks were overlaid to reveal positions with enriched peak density, indicating a transcription–replication collision (TRC).

### Proximity ligation assay

Proximity ligation assay (PLA) for flow cytometry: 500 thousand HAP1 cells were seeded in a six-well plate format 24 h prior to harvesting. Cells were trypsinized, collected, washed once with PBS, and pre-extracted using PBS-0.5% Triton for 10 min on ice. Cells were then fixed permeabilized for 15 min with BD Cytofix/Cytoperm (BD bioscience) at room temperature. PLA was performed according to the manufacturer’s protocol (DUO94001). Cells were blocked using blocking solution at 37°C for 60 min and incubated with primary antibodies (Supplementary Table S3) at room temperature for 1 h for flow cytometry. This was followed by PLA probe incubation at 37°C for 1 h, ligation for 30 min at 37°C, and then amplification at 37°C overnight. The next day, samples were washed with wash buffer (DUO82047) and incubated with detection solution at 37°C for 30 min. Cells were resuspended in PBS with 1 μg/ml DAPI supplemented with 250 μg/ml RNase A. Samples were acquired using A5 FACS Symphony (BD Biosciences) and analysed with FlowJo v.10.10.0. Single-cell measurements of the PLA signal (Red) were exported and plotted using R following subtracting the background signal from the no-antibody sample.

PLA for immuneflourescnce: 10 000 U2OS cells were seeded in 96-well imaging plates. Twenty-four later, cells were pre-extracted with ice-cold CSK buffer (10 mM HEPES, pH7.4, 300 mM sucrose, 100 mM NaCl, 3 mM MgCl_2_) supplemented with 0.5% Triton X-100 for 10 min on ice prior to fixation. The PLA assay was then performed according to the manufacturer’s protocol (DUO92013) with anti-PCNA and anti RNAPII (Supplementary Table S1). Following PLA, cells were counterstained with 1 µg/ml DAPI at room temperature and then washed with PBS × 1. Plates were imaged with an Opera Phenix spinning disc confocal microscope (PerkinElmer) at 40× magnification. Data were imported into Harmony image analysis software (PerkinElmer) for subsequent analyses.

### Animal studies

All experiments were performed with Project License PP8090463 in accordance with Home Office regulatory guidelines. NOD SCID gamma mice were obtained from Charles River Laboratories, kept in pathogen-free conditions on a 12 h light: dark cycle, and allowed to acclimatize for a minimum period of 7 days prior to surgery (∼9 weeks old). Mice were group housed (5 mice/cage) with a room temperature of 22°C ± 2°C and humidity of 55% ± 10%, provided with drinking water ad libitum and fed on a maintenance diet (PicoLab) with environment enrichment added.

### HAP1 cell-derived mouse xenograft model

WT, *CDK8* KO, *CDK8/HDA3* dKO, or *CCNC* KO HAP1 cells were prepared in Matrigel GFR:PBS (50:50) and injected (under a brief period of inhalatory anaesthesia) at a density of 2.5 × 10^6^ cells/100 µl/mouse, subcutaneously into the left flank. Mice were monitored for weight loss post-injection for the first week and then twice weekly thereafter as a minimum. Mice were also frequently assessed for signs of tumour growth and once established, the width and breadth of the tumours were measured (using callipers) to calculate the surface area (cm^2^) of a tumour. This was performed three times weekly (minimum) to ascertain tumour growth kinetics. Tumour sizes were calculated by multiplying the maximum length by the maximum breadth. When the average tumour size (per study cohort) reached ∼0.45 cm^2^, mice were assigned into treatment cohorts using a stratified randomized approach, aiming to evenly distribute tumour sizes. Sizes were ranked from low to high and assigned to a cohort using a spiral approach (*n* = 8 mice for each group). Each group received either control (Vehicle) or AZD6738 25 mg/kg orally once daily. Mice were euthanized using a Schedule 1 method (cervical dislocation) followed by a secondary confirmation of death. AZD6738 was formulated in 10% (w/v) DMSO, 40% (w/v) PEG-300, and 50% deionized water.

### Statistical analysis of xenograft tumour data

Tumour area measurements were normalized to values on day 12, which marked the initiation of treatment. To quantify tumour progression, the area under the curve (AUC) was calculated for each subject. Two sets of AUCs were derived: pre-treatment AUCs (AUC1) (days 0–12), and treatment-period AUCs (AUC2) (day 12 until study endpoint). Group comparisons of AUCs were performed. All analyses were performed using GraphPad Prism.

## Results

### CRISPR screens identify epigenetic vulnerabilities that overcome ATRi resistance

**Figure 1.**
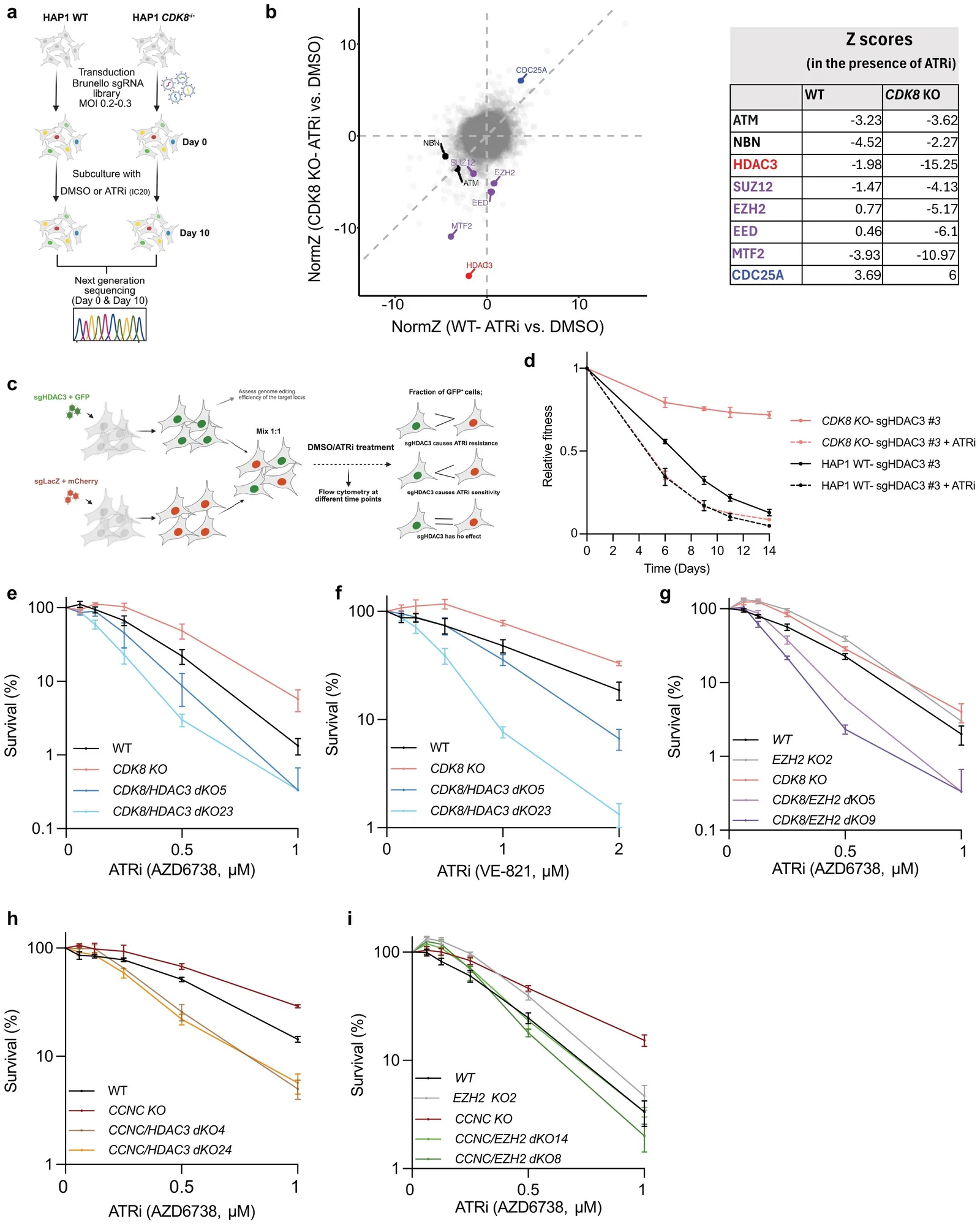
Loss of HDAC3 or PRC2 enhances ATRi efficacy in *CDK8*/CCNC-deficient cells. (**a**) Layout of the CRISPR-Cas9 screens to identify factors affecting ATRi efficacy. (**b**) Left: genome-wide CRISPR-screen results showing NormZ scores in *CDK8* WT (*x*-axis) and *CDK8* KO (*y*-axis) HAP1 cells in response to ATRi (AZD6738). Right: NormZ scores for the indicated genes. (**c**) Schematic of two-colour competitive growth assay. (**d**) Competitive growth assay results in *CDK8* WT or *CDK8* KO cells using sgRNA targeting HDAC3 coupled with GFP, as well as a sgRNA targeting LacZ coupled with mCherry. Populations were treated with indicated concentrations of ATRi (AZD6738) or DMSO throughout. Plotted are fractions of GFP-positive cells normalized to day 0. Error bars represent SEMs of three biologically independent experiments. alamarBlue cell viability assay of WT, *CDK8* KO, and *CDK8/HDAC3* dKO cells (clones #5 and #23) upon treatment with ATRi; AZD6738 (**e**) or VE-821 (**f**). (**g**) alamarBlue cell viability assay of WT, *EZH2* KO2, *CDK8* KO, and *CDK8/EZH2* dKO cells (clones #5 and #9) upon treatment with ATRi (AZD6738). (**h**) alamarBlue cell viability assay of WT, *CCNC* KO, and *CCNC/HDAC3* dKO cells (clones #4 and #24) upon treatment with ATRi (AZD6738). (**i**) alamarBlue cell viability assay of WT, *EZH2* KO2, *CCNC* KO, and *CCNC/EZH2* dKO cells (clones #8 and #14) upon treatment with ATRi (AZD6738). The same values of *EZH2* KO2 survival were used in Fig. 1g and i. For all panels, data are represented as the mean ± SEM (*n ≥ *3).

To explore mechanisms underlying ATRi sensitivity and resistance, we conducted genome-wide CRISPR-Cas9 genetic screens in an isogenic pair of *CDK8* WT and ATRi-resistant *CDK8* KO human HAP1 cells [20, 33] (Fig. 1a). Each cell line was treated with DMSO or the ATRi AZD6738 (Ceralasertib) at an IC20 dose (inhibitory concentration causing 20% reduction in cell number over 10 days). Relatively low ATRi doses were chosen on the premise that this would enhance prospects for discovering genes/pathways that, when lost, caused ATRi hypersensitivity.

Ensuing DrugZ bioinformatics analyses comparing sgRNA counts in ATRi-treated cells with those from DMSO-treated cells identified previously reported factors and pathways affecting ATRi sensitivity in WT cells, such as ATM and NBN (Fig. 1b). The data also indicated that these genes had similar impacts in the context of *CDK8* deficiency. Furthermore, our analyses identified genes whose deficiencies are known to cause resistance to ATRi in WT cells, such as CDC25A [34]. Importantly, CDC25A loss also had a strong effect on resistance in *CDK8* KO cells (Fig. 1b), a phenotype consistent with previously published data [20], and implying that CDK8 and CDC25A drive ATRi-resistance via distinct mechanisms. Overall, these results served as positive controls, indicating the reliability of our screening approach.

Importantly, we identified notable differences in gene dropouts between the *CDK8* KO and *CDK8* WT cell backgrounds (Fig. 1b). One of the strongest dropouts in *CDK8*-deficient cells was HDAC3, encoding histone deacetylase 3 (normZ score -15.25). By contrast, sgRNAs targeting HDAC3 were only mildly depleted in *CDK8*-proficient cells (normZ score -1.98; Fig. 1b). Additionally, core subunits of the PRC2 – EZH2, EED and SUZ12 – and a PRC2-associated protein, MTF2 that recruits PRC2 complex to DNA [35], selectively dropped out in *CDK8*-deficient cells upon ATRi treatment. These data suggested that loss of either *HDAC3* or the PRC2 complex would resensitize *CDK8* KO cells to ATRi.

Analysis of results from our screens conducted in the absence of ATRi, indicated that HDAC3 loss slightly impaired proliferation of WT cells, but not *CDK8*-deficient cells, suggesting a context-specific dependency (Supplementary Fig. S1a). We also observed that CDK19, a CDK8 paralogue, dropped out as a sensitizing hit in *CDK8*-deficient but not *CDK8*-proficient cells (Supplementary Fig. S1a). This is consistent with previous findings indicating that CDK8 and CDK19 function redundantly to support Mediator function and cell proliferation [36].

To validate the effect of epigentic regulators on ATRi sensitivity, we first performed two-colour competitive cell growth assays. Thus, Cas9-expressing *CDK8* KO and WT HAP1 cells were transduced with lentiviral particles of sgRNA targeting HDAC3 and GFP (green fluorescent protein), or a LacZ-targeting sgRNA and mCherry. GFP-labelled HDAC3-targeted cells were mixed in equal proportions with mCherry-labelled control cells, cultured with or without ATRi, and the relative abundance of GFP- and mCherry-expressing cells monitored over two weeks by flow cytometry (Fig. 1C). Ensuing analyses showed (for two different sgRNAs targeting HDCA3) that HDAC3 loss strongly hypersensitized *CDK8* KO cells to ATRi but had a much lesser impact in WT cells (Fig. 1d and Supplementary Fig. S1b). Furthermore, HDAC3 loss led to a strong fitness disadvantage in WT cells (Fig. 1d and Supplementary Fig. S1b), consistent with its essential role in embryonic development [37]. Importantly, the fitness disadvantage caused by HDAC3 loss was largely lost in *CDK8* KO cells, an observation that recapitulates results of the CRISPR screen (Supplementary Fig. S1a).

Although HDAC3 is generally considered to be essential for cell viability/proliferation, we were able to generate *HDAC3* KO HAP1 cells in both *CDK8*-deficient and proficient settings (Supplementary Fig. S1c). As shown in Supplementary Fig. S1d, while *CDK8* KO cells proliferated slightly slower than WT cells, *HDAC3* KO cells proliferated substantially slower, with this phenotype being partially rescued in *CDK8/HDAC3* double KO (dKO) cells. These observations are in line with our CRISPR screen results, showing that HDAC3 loss is better tolerated in *CDK8* KO cells compared to WT cells (Supplementary Fig. S1a). Through cell viability assays, we observed that while *CDK8* KO cells were more resistant than WT cells to the ATRi, AZD6738, *CDK8/HDAC3* dKO clones were more sensitive than WT cells to this compound (Fig. 1e). We obtained similar results with the structurally different ATRi, VE-821 (Fig. 1f), showing that the effect of HDAC3 loss applies generally to ATRi, and is unlikely to result from any off-target effects of the compounds used.

To validate the effect of the PRC2 complex on ATRi sensitivity, we depleted the PRC2 catalytic subunit EZH2 (Supplementary Fig. S1e). Through cell viability assays, we observed that while *EZH2* KO cells were slightly resistant to ATRi, *CDK8/EZH2* dKO cells were hypersensitive to ATRi (Fig. 1g).

Since CDK8 functions in association with CCNC, and CCNC depletion also confers ATRi resistance [20], we assessed whether loss of HDAC3 or PRC2 components re-sensitized *CCNC* KO cells to ATRi. Thus, we generated *CCNC KO, CCNC/HDAC3* dKO, *CCNC/EZH2* dKO, HAP1 cell lines (Supplementary Fig. S1e–g) and observed that *CCNC/HDAC3* dKO or *CCNC/EZH2* dKO cells were hypersensitive to ATRi when compared to *CCNC* KO cells (Fig. 1h and i). Collectively, these findings underscored HDAC3 and PRC2 components as being synthetic vulnerabilities to ATR inhibition in *CDK8/CCNC*-deficient contexts.

### Pharmacological inhibition of HDAC3 and EZH2 phenocopies their genetic loss

**Figure 2.**
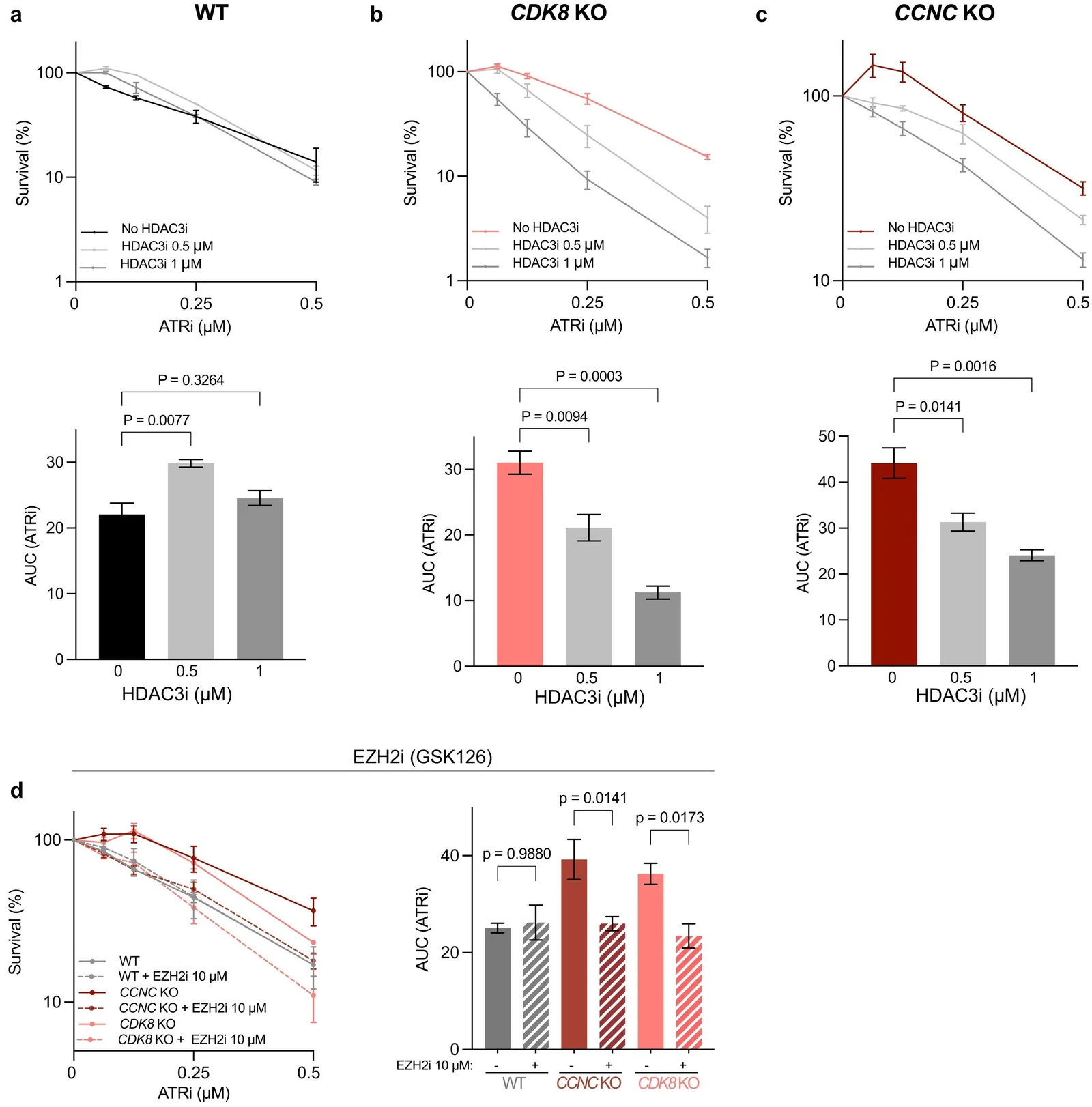
Pharmacological inhibition of HDAC3 or EZH2 hypersensitizes *CDK8*/*CCNC* KO cells to ATR inhibition. alamarBlue cell viability assay of WT (**a**), *CDK8* KO (**b**), and *CCNC* KO (**c**) HAP1 cells upon treatment with ATRi only or in combination with increasing concentrations of HDAC3 inhibitor (HDAC3i) (RGFP966). Error bars represent means ± SEM from at least three biologically independent experiments. Data represented as AUCs. (**d**) alamarBlue cell viability assay of the indictaed cell lines upon treatment with ATRi only or in combination with EZH2i (GSK126). Error bars represent means ± SEM from three biologically independent experiments. Data represented as AUCs. Statistical analyses were performed using one-way analysis of variance (ANOVA) test.

To investigate whether loss of HDAC3 could be phenocopied via inhibiting it pharmacologically, we performed cell survival studies in WT, *CDK8* KO, and *CCNC* KO cells with a combination of ATRi and a specific HDAC3 inhibitor (HDAC3i) (RGFP966). We chose a range of HDAC3i concentrations that, when used alone, did not significantly affect cell viability (Supplementary Fig. S2a). This work showed that, in WT cells, the combined treatment of ATRi and HDAC3i had little/no effect when compared with each inhibitor alone (Fig. 2a). In contrast, we observed a greater, dose-depedent combined effect in *CDK8* KO and *CCNC* KO HAP1 cells (Fig. 2b and c). To extend our findings, we tested the combined effect of ATRi and HDAC3i in other cancer cell lines including the human osteosarcoma cell line U2OS, the human prostate cancer cell lines C42 and LNCaP, and the human colorectal cancer cell line DLD1. In these cell lines, we observed that loss of either CDK8 or CCNC made cells resistant to ATRi, while combined treatment with HDAC3i restored sensitivity to ATRi (Supplementary Fig. S2b–e). These results support a model in which the effect of HDAC3 in modulating the response of *CDK8*- and *CCNC*-deficient cells to ATRi is not cell line specific and, at least in part, depends on its catalytic activity. To further substantiate this, we complemented *CDK8*/*HDAC3* and *CCNC*/*HDAC3* dKO HAP1 cell lines with vectors expressing WT or a HDAC3 deacetylase inactive mutant (Y298F) [38] (Supplementary Figs S2c and S3a). Cell survival assays showed that while complementation with WT HDAC3 rescued ATRi sensitivity, the catalytically inactive mutant failed to do so in both *CDK8/HDAC3* and *CCNC/HDAC3* dKO backgrounds (Supplementary Fig. S3d and e).

We next tested the effect of PRC2 complex inhibition on ATRi sensitivity. Inhibiting the methyltransferase activity of EZH2 using two different EZH2 inhibitors (EZH2i) or disrupting the PRC2 complex with the EED-targeted PROTAC [39], re-sensitized *CDK8* KO and *CCNC* KO cells to ATRi, but had no apparent effect on WT cells (Fig. 2d and Supplementary Fig. S4).

To investigate the functional relationship between HDAC3 and PRC2 in the context of re-sensitizing *CDK8/CCNC* KO cells to ATR inhibition, we assessed cell viability following individual or combined loss/inhibition of these complexes upon ATRi treatment. Notably, in the absence of HDAC3, EZH2 inhibtion did not further increase the ATRi sensitivity of *CDK8* or *CCNC* KO cells (Supplementary Fig. S5a and b). Conversly, HDAC3 inhibtion did not further sensitize *CCNC/EZH2* dKO cells to ATRi (Supplementary Fig. S5c). These results indicate an epistatic functional interaction between HDAC3 and PRC2 in maintaining the viability of *CDK8/CCNC* deficient cells during ATR inhibition. To determine whether this epistatic relationship reflects a biochemical dependency, we performed H3K27me3 ChIP-seq across the different genetic backgrounds. Indeed, we found—consistent with this epigentic mark’s association with transcription repression—a sharp dip in the H3K27me3 signal at TSS across all tested cell lines [40], whereas *CDK8/HDAC3* dKO cells exhibited a greater drop in H3K27me3 occupancy at promoters where RNAPII signal had been enriched according to our RNAPII ChIP-seq when compared to *CDK8* KO cells (Supplementary Fig. S5d). Together, these approaches indicated that HDAC3 and PRC2 function within a common pathway to support the viability of CDK8/CCNC-deficient cells during ATRi treatment. Based on these findings and the robust genetic interaction between HDAC3 and CDK8/CCNC loss that we observed in our screen and validation assays, we selected HDAC3 for further mechanistic investigations.

### HDAC3 loss induces replication stress and apoptosis in ATRi-treated *CDK8*/*CCNC* KO cells

**Figure 3.**
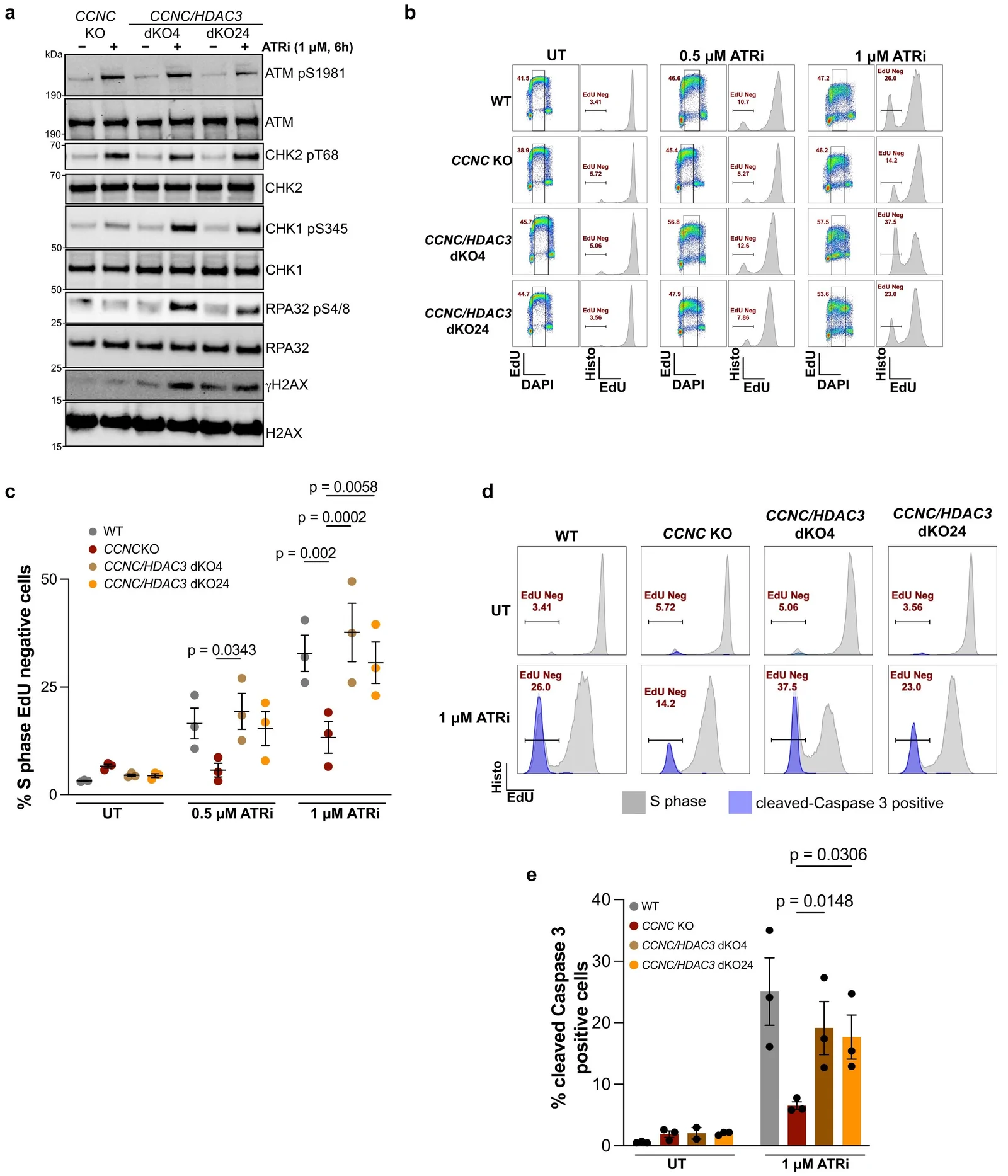
Loss of HDAC3 induces replication stress and apoptosis in *CCNC* KO cells upon ATRi. (**a**) Western blot analyses of replication stress and DSB formation markers. *CCNC* KO and *CCNC/HDAC3* dKO HAP1 cells were treated for 6 h with 1 μM ATRi prior to lysis. Samples were obtained from the same experiment but run on separate gels. One gel was used to detect pATM, pCHK1, pRPA32, and γH2AX; a second gel for ATM, CHK1, RPA32, and H2AX; a third gel for pCHK2; and a fourth gel for CHK2. Data are representative of at least four independent experiments. (**b**) Representative flow cytometry plots of WT, *CCNC* KO, and *CCNC/HDAC3* dKO clones (#4 and #24) cells following 24 h treatment with ATRi, showing total cell populations at the different cell cycle phases using EdU versus DAPI intensity (grey). Histogram plots of S-phase cells are shown measuring percentage of EdU negative (EdU Neg) cells. (**c**) Quantification of the percentage of S-phase EdU negative cells from three independent experiments. Data are represented as mean ± SEM. Statistical analyses were performed using two-way ANOVA test. (**d**) Representative histograms of WT, *CCNC* KO, and *CCNC/HDAC3* dKO clones (#4 and #24) cells following 24 h treatment with ATRi. Histograms show total cell population as separated by EdU negative and positive cells (grey) overlaid with cleaved-Caspase3 positive cells (blue). (**e**) Quantification of the percentage of cleaved-Caspase3 positive WT, *CCNC* KO, and *CCNC/HDAC3* dKO cells following ATRi treatment from three independent experiments. Data are represented as mean ± SEM. Statistical analyses were performed using two-way ANOVA test.

To explore how HDAC3 loss resensitizes *CDK8*- or *CCNC*-deficient cells to ATR inhibition, we assessed markers of replication stress and DNA damage. *CDK8/HDAC3* and *CCNC/HDAC3* dKO cells exhibited elevated ATRi-induced phosphorylation of CHK1, RPA32, and histone H2AX compared to their respective single gene KO counterparts (*CDK8* KO or *CCNC* KO) (Fig. 3a and Supplementary Fig. S6a), indicating increased ATRi-induced replication stress and DNA damage. However, phosphorylation of ATM and its target, CHK2, were not appreciably affected, suggesting that the ATRi-sensitivity in dKO cells was not likely related to ATM pathway activation following DNA damage induction.

To investigate the potential consequences of HDAC3 loss on DNA replication dynamics, we labelled cells with EdU (5-ethynyl-2′-deoxyuridine), a thymidine analogue incorporated into newly synthesized DNA, then stained them with DAPI to measure total DNA content, and used flow cytometry to identify and quantify cells in S phase and assess their DNA replication activity. Under unperturbed conditions, *CCNC/HDAC3* dKO cells exhibited a higher proportion of EdU-positive cells compared to *CCNC* KO cells, (Supplementary Fig. S6b and c), indicating possible replication stress and delayed S-phase progression in *CCNC*/*HDAC3* dKO cells. Furthermore, upon ATRi treatment, we observed a marked increase in the percentage of EdU-negative cells with S-phase DNA content among *CCNC/HDAC3* dKO cells relative to *CCNC* KO cells (Fig. 3b and c).

The presence of EdU-negative (i.e. non-replicating) S-phase cells, suggested that ATRi treatment may prevent completion of DNA replication. This is consistent with earlier work demonstrating that ATR kinase activity is essential for maintaining replication during S phase by preventing exhaustion of replication resources [41]. Indeed, the EdU-negative S-phase cells stained positive with an antibody against cleaved Caspase 3, a marker of apoptosis (Fig. 3d), suggesting that apoptosis was initiated in cells experiencing replication-associated stress. Moreover, *CCNC/HDAC3* dKO cells showed significantly higher levels of apoptosis following ATRi treatment compared to *CCNC* KO cells, which is in line with their increased sensitivity to ATR inhibition (Fig. 3d and e).

Taken together, these findings indicated that HDAC3 loss exacerbates replication stress and DNA damage in *CDK8*- or *CCNC*-deficient cells, impairs completion of DNA replication, and leads to S-phase-specific apoptosis upon ATR inhibition.

### HDAC3 loss alters RNAPII genomic distribution and promotes transcription–replication conflicts

Replication stress frequently arises from transcriptional perturbations and conflicts between the transcription and replication machinery. Consistently, our previous work demonstrated that loss of CDK8 or CCNC confers resistance to ATRi through suppressing transcription-associated replication stress [20]. Building on this, we next investigated whether HDAC3 loss alters transcription in a way that could explain the heightened ATRi sensitivity of *CDK8/CCNC*-deficient cells. HDAC3 regulates histone acetylation and mediates RNAPII transcriptional repression through forming a complex with the SMRT/N-CoR corepressor [42–45], raising the possibility that its loss promotes transcriptional dysreglation. To test this, we first examined the impact of HDAC3 loss on R-loops, RNA:DNA hybrid structures formed co-transcriptionally that can impede replication and trigger genome instability. Although R-loops serve important cellular functions, including roles in immunoglobulin class-switch recombination, telomere homeostatis, chromosome segregation, and gene expression, when not properly regulated, they can also pose threats to genome stability [46–48].

**Figure 4.**
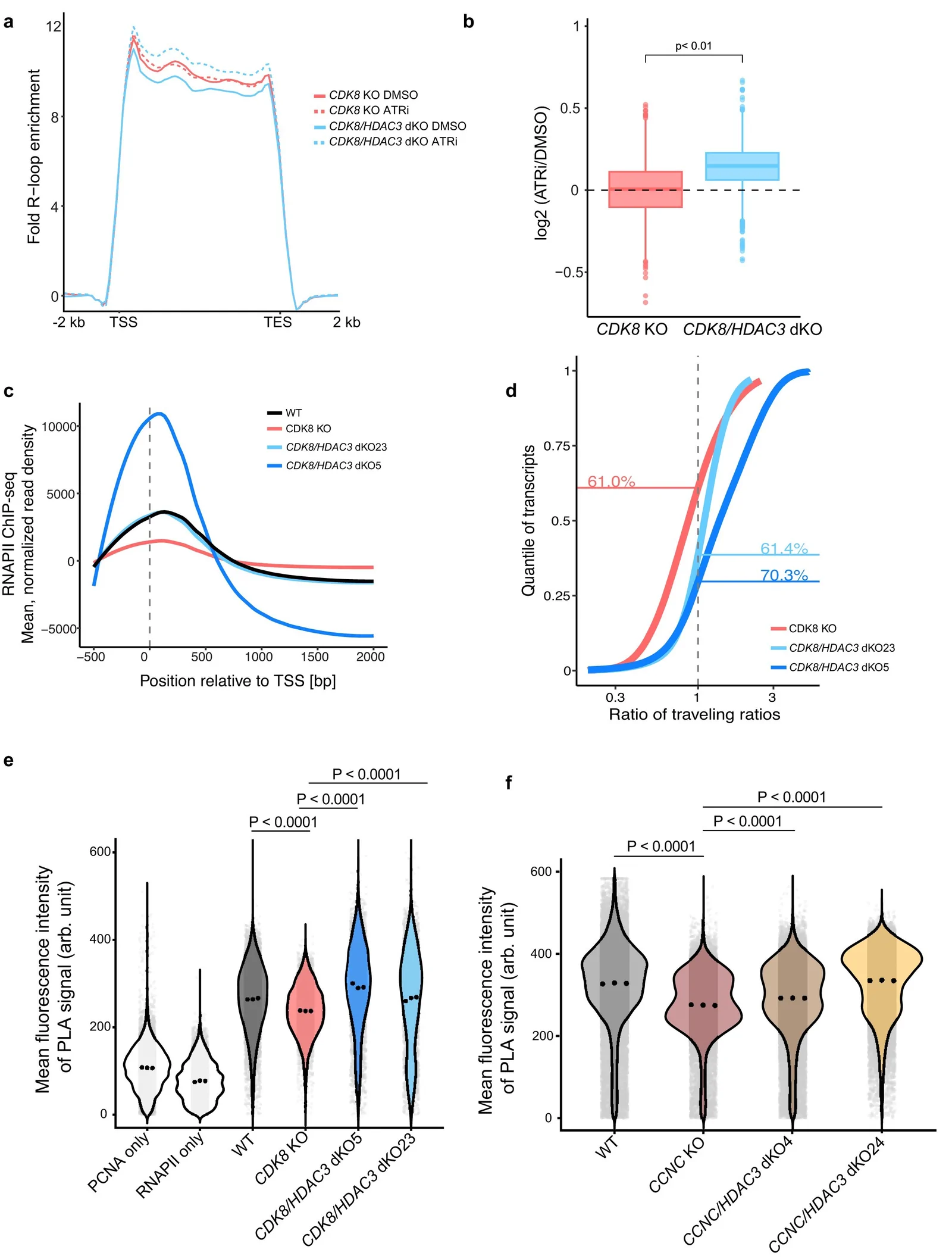
HDAC3 depletion changes RNAPII dynamics in *CDK8* KO cells. (**a**) Metagene plot of the distribution profile of S9.6 signals along all genes and flanking regions (±2 kb) in *CDK8* KO (red) and *CDK8/HDAC3 dKO23* (blue) HAP1 cells treated with either DMSO or ATRi. (**b**) Boxplots showing the log_2_ ratio of DRIP signal between ATRi-treated and DMSO conditions for *CDK8* KO and *CDK8/HDAC3* dKO23 HAP1 cells. The box ends represent upper and lower quartiles, the centre line represents median and the whiskers extend to 1.5 times the interquartile range beyond the box. Statistical analyses were performed using two-sided, unpaired Wilcoxon test. (**c**) RNAPII ChIP-seq profile in WT, *CDK8* KO, and *CDK8/HDAC3* dKO clones (#5 and #23) (biological *n* = 4). TSS, transcription start site. (**d**) Ratios of traveling ratio of RNAPII in *CDK8* KO and *CDK8/HDAC3* dKOs (#5 and #23), relative to WT cells (biological *n* = 4). (**e, f**) Violin plots represent the mean fluorescence intensity of PLA signal, as measured by flow cytometry, in the indicated cell lines. Black circles represent means of three independent biological replicates. Statistical analyses were performed using using one-way ANOVA test with multiple comparisons.

By performing DRIP-seq (DNA–RNA immunoprecipitation followed by deep sequencing) with the S9.6 monoclonal antibody for genome-wide mapping of R-loops [49], we observed significantly higher ATRi-induced levels of R-loops in *CDK8/HDAC3* dKO cells compared to *CDK8* KO control cells (Fig. 4a and b). Nevertheless, overexpressing WT RNaseH1—a key enzyme involved in detecting and resolving R-loops through degrading the RNA moiety of the hybrid—resulted in only a modest, partial rescue of ATRi sensitivity in *CDK8/HDAC3* and *CCNC/HDAC3* dKOs (Supplementary Fig. S7a–c). Such partial rescue could be explained by either not fully effective resolution of stable R-loops by the overexpressed RNaseH1, or by R-loops not being the main/sole contributors to the ATRi sensitivity observed in the dKO cells. We cannot fully exclude R-loops as a contributing source, given the inherent limitations of RNaseH1 overexpression as a tool for complete R-loop resolution, as previously observed and discussed in the context of transcription-replication conflicts [50]; nonetheless, these results echo an earlier report indicating that R-loops are not directly responsible for ATRi-resistance in CCNC-depleted cells [20].

Since R-loop persistence provided only a partial explanation for our findings, we next examined whether broader transcriptional dysregulation caused by HDAC3 loss could lead to a replication stress phenotype. The observed impact of HDAC3 status on R-loops following ATRi treatment suggested that loss of HDAC3 may act upstream to exacerbate transcription–replication conflicts, contributing to replication stress and impaired genome stability. To assess this, we performed RNAPII ChIP-seq (chromatin immunoprecipitation followed by deep sequencing), and observed, consistent with previous studies [51], a substantial genome-wide decrease in RNAPII levels at TSSs in *CDK8* KO cells compared to WT controls (Fig. 4c). This decrease was reversed in *CDK8/HDAC3* dKO cells (Fig. 4c), although the level of restoration varied between dKO clone 23 and dKO clone 5, perhaps because of clonal variation. To further corroborate these findings and address the variability observed between clones, we generated two more independent *CDK8/HDAC3* dKO clones in HAP1 cells; #14 and #15 (Supplementary Fig. S7d), and performed RNAPII ChIP-seq. As shown in Supplementary Fig. S7e, the additional dKO clones exhibited increased RNAPII occupancy at TSS compared with their parental *CDK8* KO cells, consistent with the trend observed in dKO23. These data support the interpretation that the difference observed between dKO5 and dKO23 reflects clonal variation rather than a systematic genotype-dependent effect, and confirm that HDAC3 loss broadly restores RNAPII occupancy in *CDK8* KO cells.

To quantitatively evaluate how transcription was affected in the *CDK8/HDAC3* dKO cells, we calculated the RNAPII ‘traveling ratio’, which reflects the relative density of RNAPII in promoter-proximal regions compared to the gene bodies [52, 53]. In agreement with our other findings, traveling ratios were globally higher in WT and *CDK8/HDAC3* dKOs compared to *CDK8* KO cells (Supplementary Fig. S7f). Moreover, when we focused on genes that displayed an increased RNAPII peak at the TSS, we found that relative to WT cells, 60% of these genes had a decreased TR in *CDK8* KO cells. Conversely, ∼70% and ∼60% of the genes had an increased traveling ratio in dKO5 and dKO23 cells, respectively (Fig. 4d). To assess whether this reflected a global change in transcriptional activity, we measured nascent RNA synthesis by EU incorporation, which revealed a marked increase in transcriptional output in *CDK8/HDAC3* and *CCNC/HDAC3* dKO cells compared to controls (Supplementary Fig. S7g and h). Together, the increased transcriptional activity and the higher recruitment of RNAPII at the TSS in *CDK8/HDAC3* and *CCNC/HDAC3* dKO cells suggest that more RNAPII molecules are most likely being recruited to initiate active transcription in the dKO cells.

We speculated that the observed increase in RNAPII at TSS in *CDK8/HDAC3* dKO cells compared to controls could elevate the potential for transcription-replication conflicts (TRCs). To test this, we performed PLA with antibodies against RNAPII and the replication component, PCNA. In agreement with our hypothesis, under untreated conditions, physical proximity of PCNA and RNAPII was significantly reduced in *CDK8* KO and *CCNC* KO cells compared to WT cells, and this reduction was reversed upon codepleting HDAC3 (Fig. 4e and f). Similarly, in U2OS cells, CCNC deficiency was associated with decreased levels of PCNA:RNAPII PLA foci when compared to WT cells, and inactivation of EZH2 in *CCNC* KO background resulted in a significant increase in PLA foci under unperturbed conditions (Supplementary Fig. S8a and b). These results suggested that while loss of CDK8 or CCNC counteracts TRCs, codepletion of HDAC3 or EZH2 increases their occurrence.

To investigate the directionality of these conflicts, we first mapped replication origins using previously published Okazaki fragment-seq data from unchallenged RPE1 cells [29] and overlaid these data with our RNAPII ChIP-seq dataset from HAP1 cells, allowing us to infer to some extent the orientation of transcription relative to replication fork progression at sites of RNAPII pausing. This analysis revealed that in *CDK8/HDAC3* dKO cells, RNAPII binding sites were more frequently associated with HO rather than CD conflicts orientation compared to *CDK8* KO cells (Supplementary Fig. S8c). It is important to note that the replication origin data used in our analyses were derived from RPE1 cells and overlaid with RNAPII ChIP-seq data from HAP1 cells. While this cross-cell-line approach provides insights, it is not optimal, as differences in replication programmes between cell types may influence the interpretation of transcription-replication conflict mapping.

Since HO collisions are intrinsically more genotoxic than CD encounters and represent a potent activating signal for ATR [54], our findings provides a potential mechanistic explanation for the heightened ATR dependency of the *CDK8/HDAC3* dKO cells. Collectively, these findings suggested a model in which *CDK8/HDAC3* dKO cells experience increased RNAPII occupancy at TSS and alterations in transcription rates. This reshaping of RNAPII dynamics upon HDAC3 loss promotes transcriptional deregulation and exacerbates HO TRCs, providing a mechanistic basis for the replication stress and ATRi hypersensitivity observed in *CDK8/CCNC*-deficient cells.

### Loss of HDAC3 hypersensitizes *CDK8* KO cells to ATRi *in vivo*

**Figure 5.**
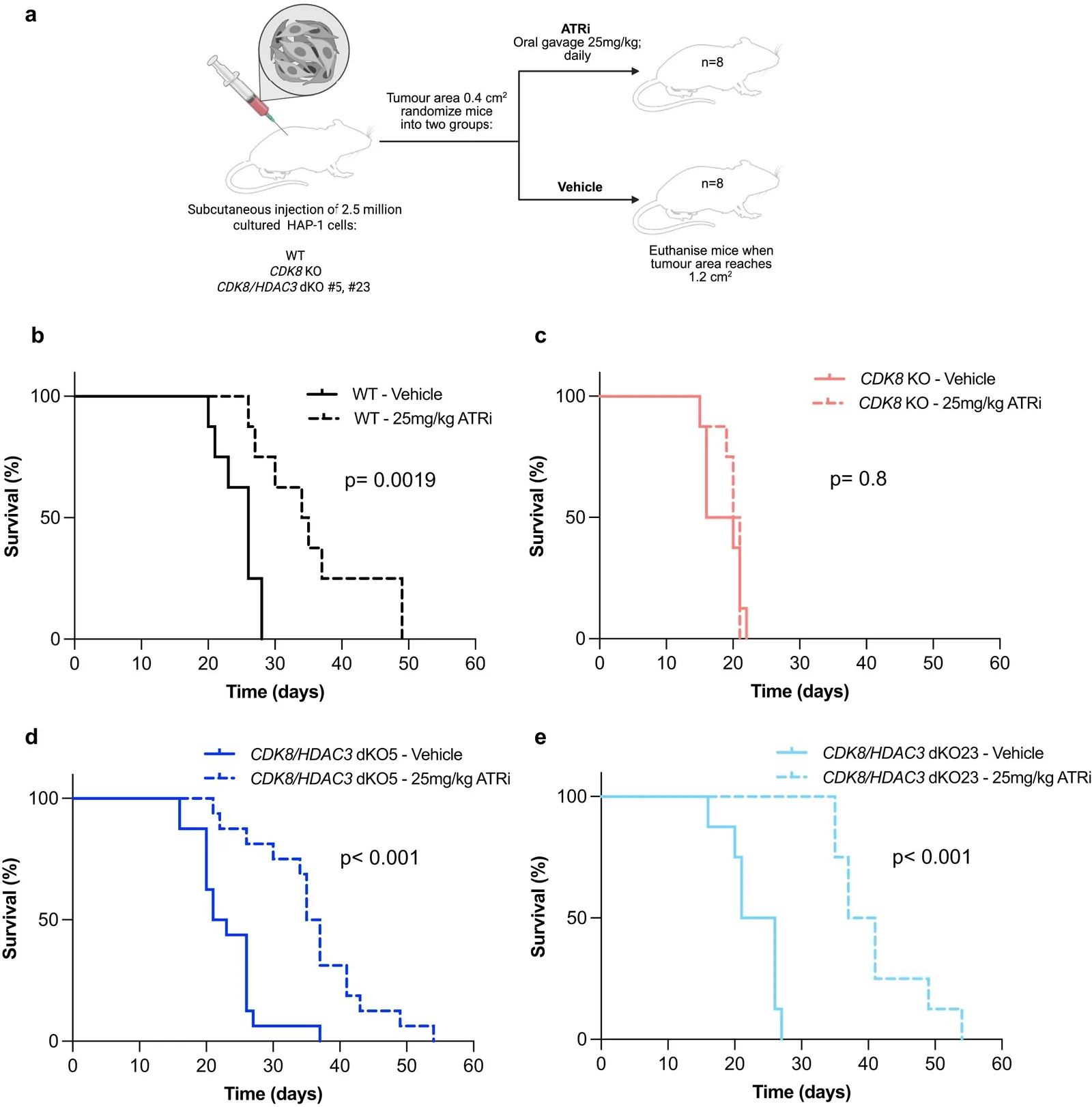
Loss of HDAC3 hypersensitizes *CDK8* KO cells to ATRi *in vivo*. (**a**) Schematic overview of the xenograft experiment. Kaplan–Meier survival curves of tumours (*n* = 8 mice per group) derived from WT (**b**), *CDK8* KO (**c**), *CDK8/HDAC3* dKO clone #5 (**d**), or *CDK8/HDAC3* dKO clone #23 (**e**) HAP1 cells treated with either ATRi (AZD6738) or vehicle. AZD6738 (25 mg/kg) was administered orally daily. Statistical analyses were performed using Mantel–Cox test.

To extend our findings into a physiological context, we assessed the impact of HDAC3 loss on ATRi efficacy in a mouse xenograft model. Thus, we implanted WT, *CDK8* KO, or *CDK8/HDAC3* dKO (clone #5 and #23) human HAP1 cells to generate tumour xenografts in mice that were randomized prior to receiving either vehicle or 25 mg/kg of ATRi AZD6738 orally, daily (Fig. 5a). Ensuing results revealed that in mice implanted with WT cells, ATRi treatment significanlty delayed tumour growth as measured by improved overall survival and tumour area (Fig. 5b and Supplementary Fig. S9a), indicating sensitivity to ATRi treatment. In contrast, ATRi had no significant effect on tumour burden or survival with tumours derived from *CDK8* KO cells (Fig. 5c and Supplementary Fig. S9b). These findings demonstrated that, relative to WT, CDK8 loss confers resistance to ATR inhibition *in vivo*. Strikingly, this resistance was reversed upon concurrent loss of HDAC3, as seen with the *CDK8/HDAC3* dKO derived tumour models, which restored ATRi sensitivity, evidenced by significantly reduced tumour area and prolonged overall survival (Fig. 5d and e; Supplementary Fig. S9c and d). Together, these findings establish that, in accord with our *in vitro* cell culture studies, loss of HDAC3 counteracts *CDK8*-driven resistance to ATRi treatment in physiological settings.

### CDK8/CCNC-mediated ATRi resistance in ARID1A- or ATM-deficient cells is counteracted by HDAC3 inhibition

Loss of ATM or ARID1A is known to confer hypersensitivity to ATR inhibition, and mutations in these genes have been proposed as biomarkers for patient selection in ATRi clinical development [19, 55–57]. Moreover, both genes are frequently downregulated in human cancers [58–61]. To assess whether our findings extend to such clinically relevant contexts, we tested whether loss of CDK8 or CCNC modulated ATRi sensitivity in ATM- or ARID1A-deficient cells.

**Figure 6.**
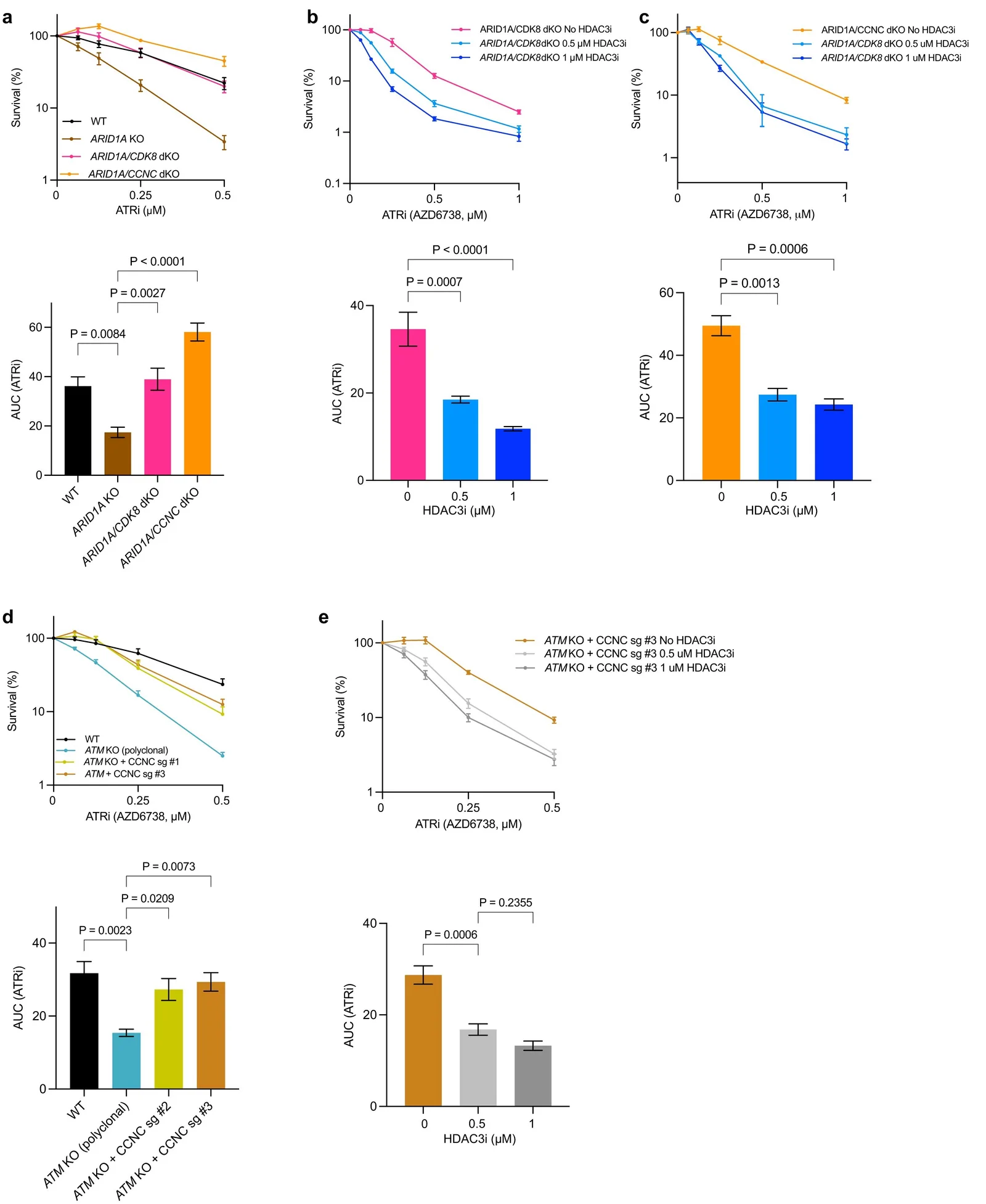
HDAC3 inhibition restores ATRi sensitivity in CDK8/CCNC-driven ATRi resistance in ARID1A- or ATM-deficient cells. (**a**) alamarBlue cell viability assay of of the indicated cell line upon treatment with ATRi (AZD6738). alamarBlue cell viability assay of ARID1A/CDK8 dKO (**b**) or *ARID1A/CCNC dKO* (**c**), HAP1 cells upon treatment with ATRi only or in combination with increasing concentrations of HDAC3 inhibitor (HDAC3i) (RGFP966). (**d**) alamarBlue cell viability assay of of the indicated cell line upon treatment with ATRi (AZD6738). (**e**) alamarBlue cell viability assay of ATM/CCNC dKO HAP1 cells upon treatment with ATRi only or in combination with increasing concentrations of HDAC3 inhibitor (HDAC3i) (RGFP966). In all panels, error bars represent means ± SEM from three biologically independent experiments. Data represented as AUCs. Statistical analyses were performed using one-way ANOVA test.

In *ARID1A*-deficient HAP1 cells, deletion of CDK8 or CCNC caused resistance to ATR inhibition (Fig. 6a). Importanlty, pharmacological inhibition of HDAC3 resotred ATRi sensitivity in both ARID1A/CDK8 and ARID1A/CCNC dKO cells (Fig. 6b and c). Similarly, we observed that loss of CDK8 rendered *ATM*-deficient HAP1 cells resistant to ATRi (Fig. 6d), which was reversed by HDAC3 inhibition (Fig. 6e).

Together, these results suggested that in cancer cells with ARID1A or ATM deficiency, which typically are responsive to ATR inhibition, the acquired loss of CDK8 or CCNC may drive ATRi resistance. Importantly, our findings suggest that in such contexts, combined HDAC3 and ATR inhibition could be used as a therapeutic strategy to overcome and exploit ATRi resistance.

## Discussion

Targeting ATR has emerged as a promising therapeutic strategy for cancers with high replication stress and/or specific genetic vulnerabilities, such as loss-of-function mutations in *TP53* [18, 19], *ATM* [14, 16–19], *ARID1A* [57], and *KLF5* [24] (reviewed in [12]). Nevertheless, in cellular models, certain genetic alterations confer ATRi resistance [12, 34, 62, 63], suggesting that this might also apply to *in vivo* settings. A key finding of our study is that ATRi resistance in the context of *CDK8*- or *CCNC*-deficiency is reversed upon loss or inhibition of epigenetic regulators such as HDAC3 or the PRC2 complex. These ‘synthetic lethal’ relationships highlight potential therapeutic opportunities for overcoming ATRi resistance in such settings.

Our findings align with previous reports linking loss of *CDK8* or *CCNC* to alleviating levels of TRCs [20], but we further reveal that their deficiency creates a dependency on the activity of HDAC3 or the PRC2 complex to sustain viability under ATRi treatment. Crucially, we demonstrate that HDAC3 plays a pivotal role in regulating transcription and RNAPII dynamics in CDK8-deficient contexts. Mechanistically, our data support a model in which these epigenetic regulators preserve replication competence at least in part by constraining RNAPII dynamics and limiting transcription–replication conflicts.

We propose that increased RNAPII density at TSSs, coupled with increased transcriptional activity (as measured by EU incorporation) upon HDAC3 loss, may heighten the probability of encounters between transcription and replication machineries, particularly at genomic loci where these processes are known to converge. Nevertheless, it is important to note that the partial rescue of ATRi sensitivity by RNaseH1 overexpression indicates that R-loops and transcription–replication conflicts, while likely contributing, may not be the sole drivers of the observed phenotype. Additional mechanisms, such as alterations in chromatin acetylation states affecting origin firing, changes in replication timing, or shifts in replication origin usage, may act in parallel to potentiate replication stress upon HDAC3 or PRC2 loss in CDK8/CCNC-deficient cells. Future work will be needed to delineate the relative contributions of these mechanisms.

Our RNAPII ChIP-seq data reveal broadly increased occupancy at TSSs CDK8/HDAC3 dKO cells, and to further investigate whether this reflects a global effect or is driven by specific gene classes, we performed additional analysis stratifying genes by length, transcriptional output, and proximity to early-replicating regions or common fragile sites—loci that are particularly prone to transcription–replication conflicts owing to the extended time required for their transcription relative to S-phase progression [64, 65]. This analysis did not reveal enrichment of RNAPII occupancy at any particular gene class or genomic feature (data not shown), suggesting that the increased transcriptional activity in CDK8/HDAC3-compromised cells reflects a broad, genome-wide effect rather than being confined to specific vulnerable loci. We therefore favour a model in which the cumulative burden of globally elevated transcription, rather than dysregulation at discrete hotspots, underlies the heightened replication stress and ATRi sensitivity observed in these cells.

We also demonstrate that the EZH2 subunit of the PRC2 complex operates in an epistatic manner with HDCA3 to maintain survival of *CDK8/CCNC* KO cells in the presence of ATRi. We speculate that both PRC2 and HDAC3 act to maintain an epigenetically inactive chromatin state through modulating methylation and acetylation states of histone H3K27, respectively, thus impacting RNAPII transcriptional activity. Consistent with this, H3K27me3 ChIP-seq analysis in *CDK8* KO and *CDK8/HDAC3* double KO cells revealed a global reduction in H3K27me3 occupancy in the double KO compared to *CDK8* KO alone, providing direct chromatin-level evidence that HDAC3 loss compromises PRC2-dependent repressive histone methylation. This further supports the biochemical interdependence between HDAC3 and PRC2 activity and reinforces the epistatic relationship observed at the phenotypic level. Indeed, it has been shown previously that HDAC3 is required for PRC2 recruitment and ensuing exclusion of RNAPII for transcriptional silencing across the X chromosome [66]. Accordingly, our results suggest an additional therapeutic opportunity for overcoming ATRi resistance in contexts of CDK8/CCNC deficiency via combining ATRi with inhibition of HDAC3 or PRC2 complex components. Nevertheless, our results show that in otherwise WT settings, loss or inhibition of EZH2 actually renders cells resistant to ATRi. This observation may be of significance for cancers carrying the H3.3K27M mutation which functionally mimics EZH2 inhibition [67–69]. We thus speculate that H3.3K27M mutated cancers are unlikely to benefit from ATR inhibition-targeted therapy.

While our data suggest that the effect of HDAC3 on ATRi efficacy in CDK8/CCNC KO cells depends on its catalytic activity, we cannot rule out the possibility that HDAC3 also exerts deacetylase-independent functions contributing to this phenotype. Future studies will be needed to dissect these catalytic versus non-catalytic contributions. Our model resonates with the findings of Lloyd *et al*. who demonstrated that RECQL5 loss resensitizes *CCNC* KO cells to ATRi [20]. Importantly, RECQL5 has been established as a global regulator of transcriptional elongation in human cells, and its loss induces RNAPII transcriptional deregulation [53]. Additionally, RECQL5 is reported to play a role in minimizing and resolving TRCs [70, 71]. These effects align with our current observations, supporting the idea that disrupting transcriptional homeostasis, whether via RECQL5, HDAC3, or PRC2 loss, creates a vulnerability to ATR inhibition.

Of note, CCNC and CDK8 display context-dependent roles in tumourigenesis [72]. While CDK8 was identified to act as an oncoprotein [73] whose gene is amplified in colorectal cancers [74], pancreatic cancers [75], and melanomas [76], it also displays tumour suppressive properties in endometrial cancers [77]. Furthermore, *CCNC* is often deleted in a subset of acute lymphoblastic leukaemias, osteosarcomas and gastric cancers [72], and cBioPortal data indicate that CCNC or CDK8 loss frequently occurs in prostate cancers and large B-cell lymphomas [59, 61, 78]. These observations indicate that loss of CCNC or CDK8 is likely to emerge in clinical settings and evoke ATRi resistance, positioning such settings—with downregulated expression or mutation of CCNC or CDK8—as prime candidates for ATRi/HDAC3i combination treatments. Notably, although HDAC3 loss is typically lethal in cells [37], we found that this lethality is rescued in CDK8- or CCNC-deficient cells, suggesting that simultaneous loss of HDAC3 and CDK8/CCNC may be tolerated in certain tumour contexts. Together, our findings raise the possibility that double loss of CDK8/CCNC and HDAC3 could arise in clinical settings and that ATR inhibition monotherapy may provide an effective means to selectively eliminate tumour cells in such settings.

### Limitations of the study

Our study supports a model in which targeting epigenetic regulators, specifically HDAC3 and the PRC2 complex, restores ATRi sensitivity in ATRi-resistant genetic backgrounds of CDK8 or CCNC deficiency, through dysregulation of RNAPII transcriptional programs and induction of high levels of transcription–replication conflicts. However, several limitations should be acknowledged to guide future research. Our mechanistic studies were performed in a subset of cell lines, and validation in more disease-relevant models will be necessary to establish the generalizability of our findings. Furthermore, the translational potential of our results remains limited in their current state, and combination treatments of ATRi/HDAC3i or ATRi/EZH2i should be evaluated *in vivo* to assess their efficacy, tolerability, and toxicity. In this regard, despite the promise of epigenetic therapies in cancer, clinical response rates remain poor, which may reflect dose-limiting toxicities that prevent therapeutic dosing. Thus, optimization of pharmacokinetics and minimization of off-tumour toxicity will be critical to achieving durable and tolerated responses in patients.

## Supplementary Material

gkag811_Supplemental_Files

## Data Availability

Sequencing data generated in this study, including NormZ scores arising from DrugZ analysis of the CRISPR-Cas9 screens, are provided in Supplementary Data 1. Datasets generated in this study have been deposited into the European Nucleotide Archive and are accessible at the accession numbers: PRJEB96732 (CRISPR-Cas9 genetic screen), PRJEB113825 (ChIP-seq), and PRJEB96733 (DRIP-seq). No original computer code was developed for this research. All analyses were conducted using standard, publicly available software packages described in the ‘Materials and methods’ section. Data, material requests, and correspondence regarding this manuscript can be directed to Samah W. Awwad (samah.diab@cruk.cam.ac.uk.) and Stephen P. Jackson (steve.jackson@cruk.cam.ac.uk.).
