# Supplementary material for: Targeting epigenetic regulators induces transcription-replication conflicts to overcome ATR inhibitor resistance
Source: Nucleic Acids Res. 2026 Aug 20;54(15):gkag811. doi: 10.1093/nar/gkag811 (PMC13490953; doi:10.1093/nar/gkag811)
Supplement: Supplementary file 1 [file gkag811_supplemental_files.zip › Supplementary data 260611.pdf]

Supplementary data for

Targeting epigenetic regulators induces transcription-replication conflicts to overcome ATR inhibitor resistance

Samah W. Awwad[1,2#], Holly Barber[1,3*], Josie Coulthard[1*], Simon Lam[1,2], Alejandra Rojas[1], Nadia Gueorguieva[1,2], Michael Woods[1,2,4], Gabriel Balmus[4,5,6], Rimma Belotserkovskaya[1,2], and Stephen P. Jackson[1,2,3#]

[1]*Cancer Research UK Cambridge Institute, University of Cambridge, Cambridge, UK*
[2]*The Gurdon Institute and* [3]*Department of Biochemistry, University of Cambridge, Cambridge, UK*
[4]*UK Dementia Research Institute and* [5]*Department of Clinical Neurosciences, University of Cambridge, Cambridge, UK.*
[6]*Department of Molecular Neuroscience, Transylvanian Institute of Neuroscience, Cluj-Napoca, Romania.*

*authors contributed equally
[#] Correspondence: samah.diab@cruk.cam.ac.uk, steve.jackson@cruk.cam.ac.uk.

This PDF file includes:

Supplementary Figures 1-9 and figure legends

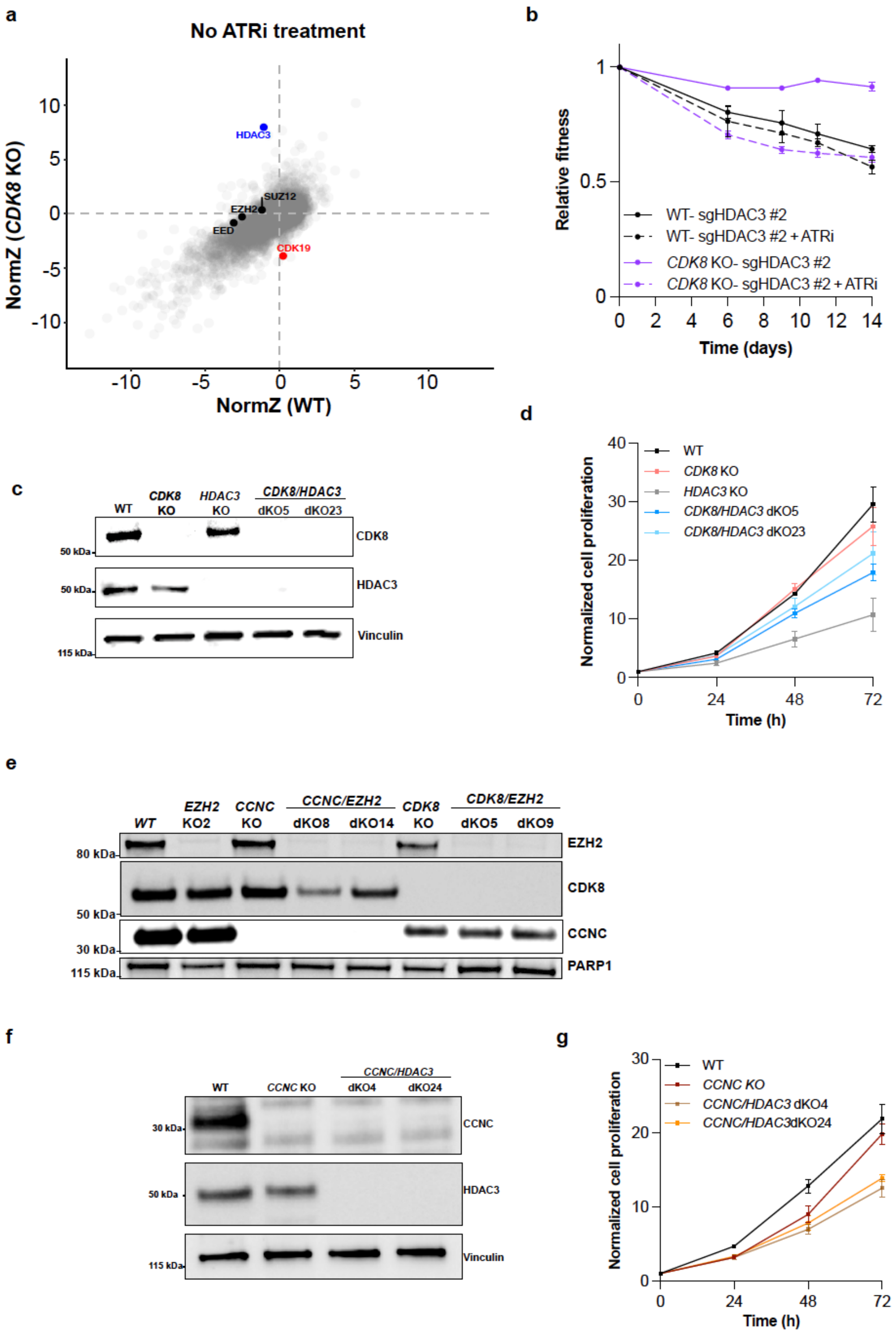

Figure S1

Supplementary Figure S1. Characterization of *CDK8*, *CCNC*, and *HDAC3* single and double knockouts. a. Genome-wide CRISPR-screen results showing NormZ scores in *CDK8* WT (x-axis) and *CDK8* KO (y-axis) HAP1 cells under DMSO-treated conditions. **b.** Competitive growth assay results in *CDK8* WT or *CDK8* KO cells expressing GFP-coupled sgRNA targeting **HDAC3** (sgRNA #2) and RFP-coupled sgRNA targeting **lacZ** (control). Cells were treated with ATR inhibitor (AZD6738) or DMSO. Plotted are fractions of GFP-positive cells normalized to day 0. Data represent mean ± SEMs of three biologically independent

experiments. **c.** Western blot analysis of CDK8 and HDAC3 expression in the indicated cell lines. Vinculin served as a loading control. **d.** Proliferation assay of WT, *CDK8* KO, *HDAC3* KO, or *CDK8/HDAC3* dKO clones (5 and 23). Cells were seeded in 96-well plates, and alamarBlue fluorescence was used as a proxy for proliferation. Values were normalised to the 0 h timepoint. Data represent mean ± SEM from three independent biological replicates. **e.** Western blot analysis of EZH2, CDK8 and CCNC expression in the indicated cell lines. PARP1 was used as loading control. **f.** Western blot analysis of CCNC and HDAC3 expression in the indicated cell line. Vinculin was used as a loading control. **g.** Proliferation assay as in (d), using WT, *CCNC* KO, *CCNC/HDAC3* dKO (clones #4 and #24).

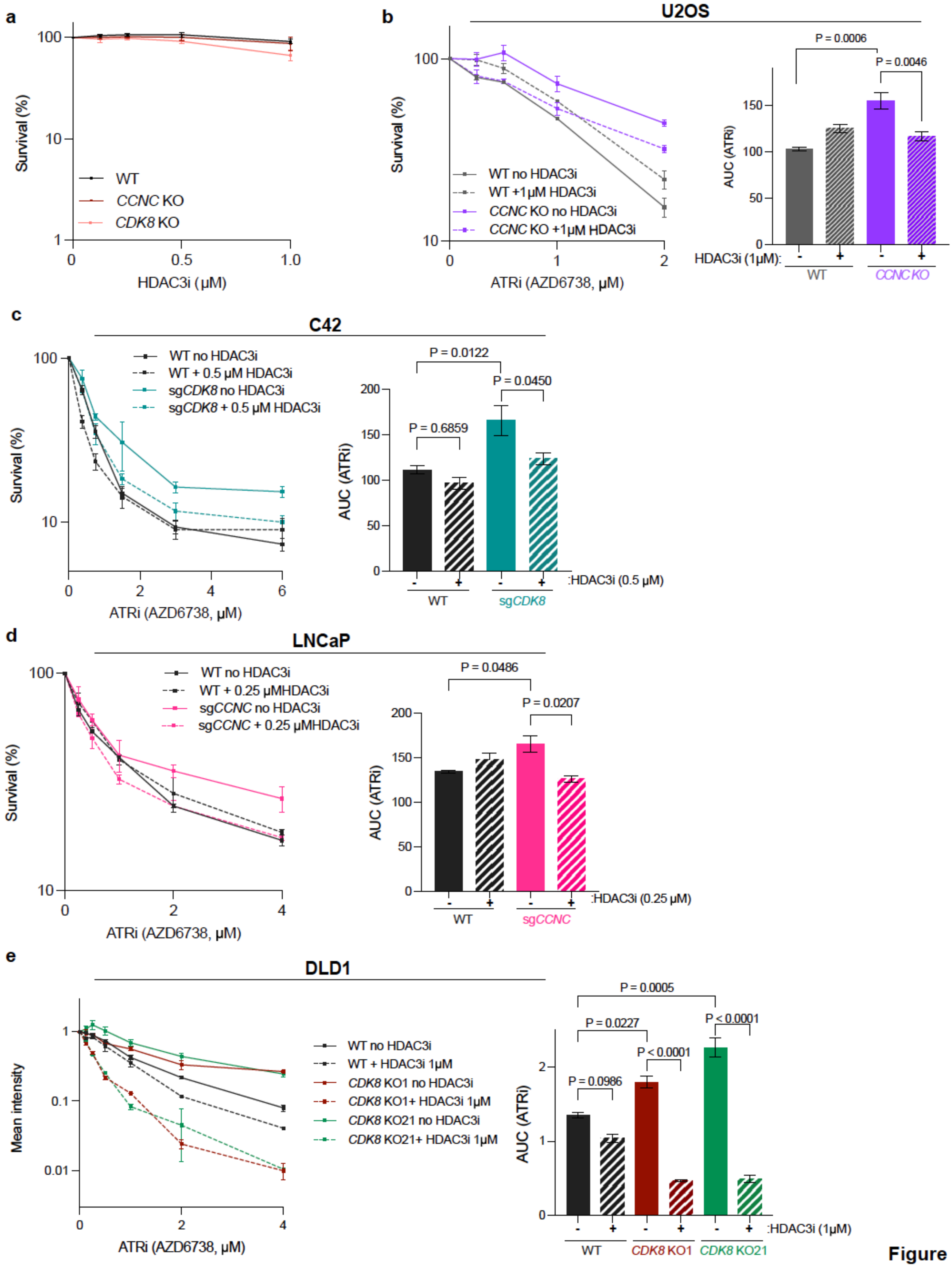

Figure S2

Supplementary Figure S2. ATRi and HDAC3i exhibit synergistic cytotoxicity in CDK8/CCNC-deficient cells across multiple cell line models. a. alamarBlue cell viability assay of WT, *CDK8* KO and *CCNC* KO cells upon treatment with HDAC3 inhibitor (RGFP966). Data represent means ± SEM from three biologically independent experiments. **b.** alamarBlue cell viability assay of WT or *CCNC* KO U2OS cells upon treatment with ATRi alone or in combination with 1 μM HDAC3i (RGFP966). **c.** alamarBlue cell viability assay of WT or sg*CDK8*-infected C42 cells upon treatment with ATRi alone or in combination with 0.5 μM

HDAC3i (RGFP966). **d.** alamarBlue cell viability assay of WT or sg*CCNC*-infected LNCaP cells upon treatment with ATRi alone or in combination with 0.25 μM HDAC3i (RGFP966). **e.** Clonogenic cell survival assay of WT or two different *CDK8* KO DLD1 clones upon treatment with ATRi alone or in combination with 1μM HDAC3i (RGFP966). Data represented as area under the curves (AUCs) and as the mean ± SEM (n=3 for U2OS and C42, and n=2 for DLD1 and LNCaP). Statistical analysis was performed using one-way ANOVA with multiple comparisons.

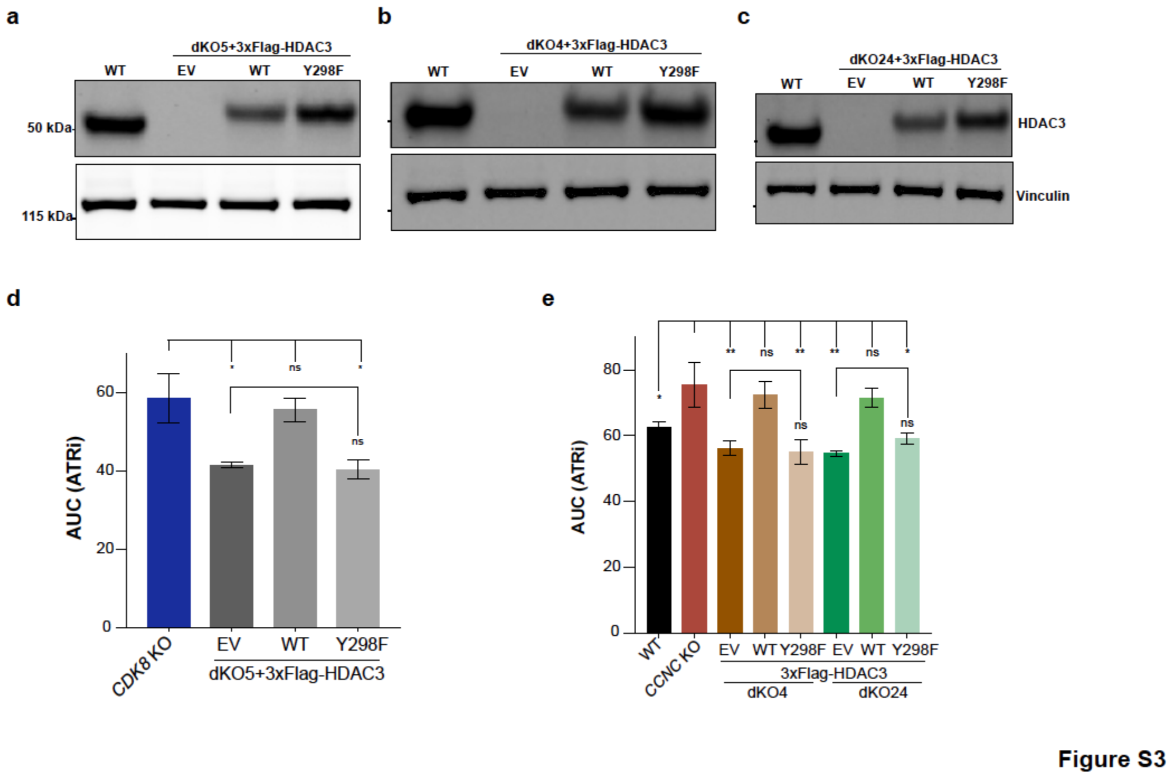

Supplementary Figure S3. HDAC3 catalytic activity is required for survival of *CDK8*- and *CCNC*-deficient cells in the presence of ATRi. a-c. Western blot analysis of HDAC3 expression in the indicated cell lines complemented with empty vector (EV), WT HDAC3 or catalytically inactive Y298F HDAC3 mutant. **d, e.** Area under the curve quantification from alamarBlue cell viability assays upon ATRi treatment in the indicated cell lines expressing EV, WT HDAC3 or Y298F mutant. Data are shown as means ± SEM from three biologically independent experiments. Statistical analysis was performed using one-way ANOVA with multiple comparisons. * $p<0.05$, ** $P<0.001$, ns = not significant.

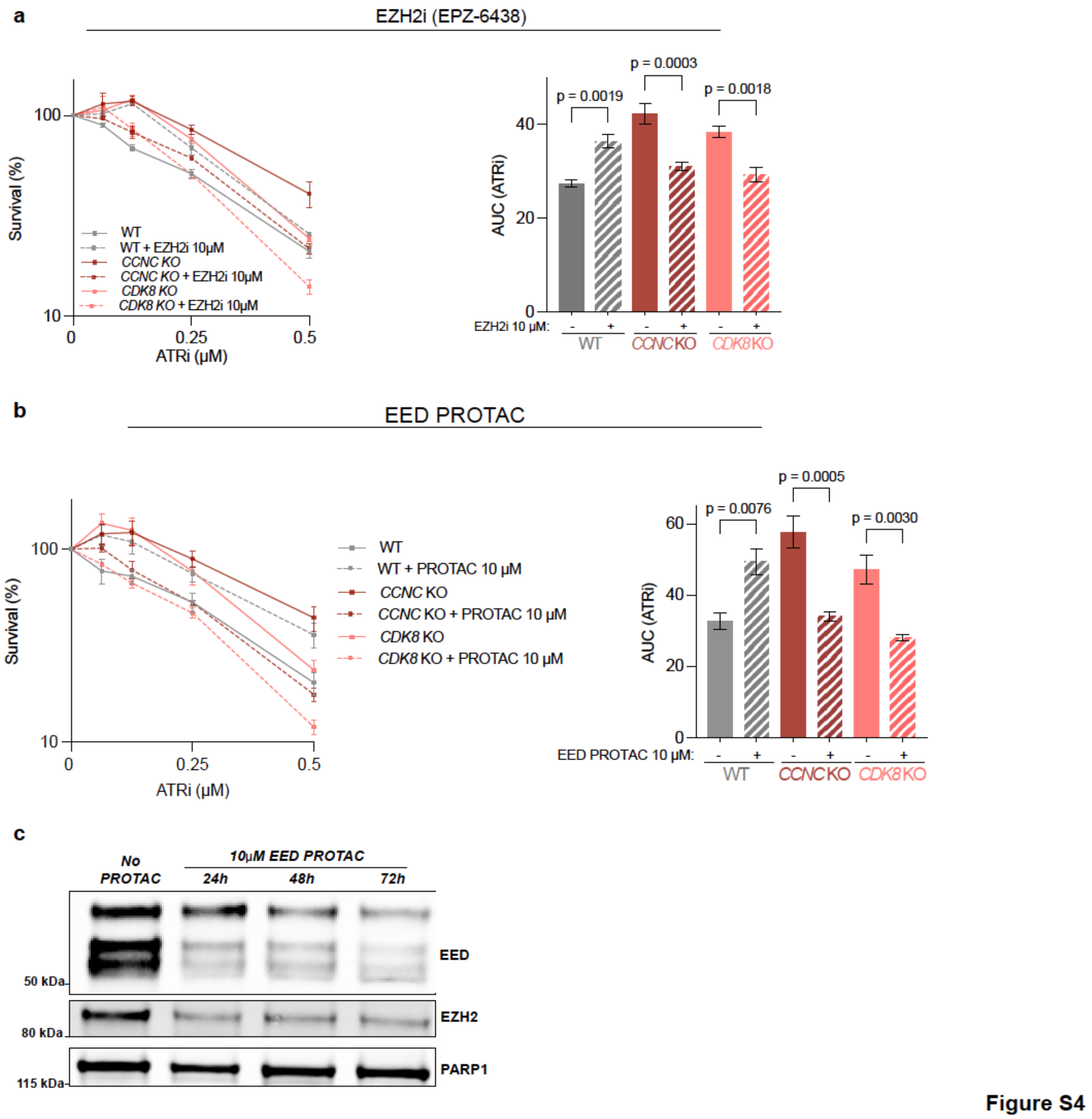

Figure S4

Supplementary Figure S4. Inhibition of PRC2 components enhances ATRi efficacy in *CDK8/CCNC* KO cells. a. alarmBlue cell viability assay of the indicated cell lines upon treatment with ATRi only or in combination with 10 µM EZH2 inhibitor (EPZ-6438)**.** Data represented as AUCs. **b.** alamarBlue cell viability assay of the indicated cell lines upon treatment with ATRi only or in combination with 10 µM EED PROTAC**.** 2500 cells/well were seeded in 96-well plates. 16 h later, cells were treated with EED PROTAC only, and ATRi was added 24 h later for 72 h. Data represented as AUCs. Error bars represent means ± SEM from three biologically independent experiments. Statistical analysis was performed using one-way ANOVA with multiple comparisons. **c.** Western blot analysis of EED and EZH2 expression upon 10 µM EED PROTAC treatment at the indicated time points. PARP1 served as a loading control.

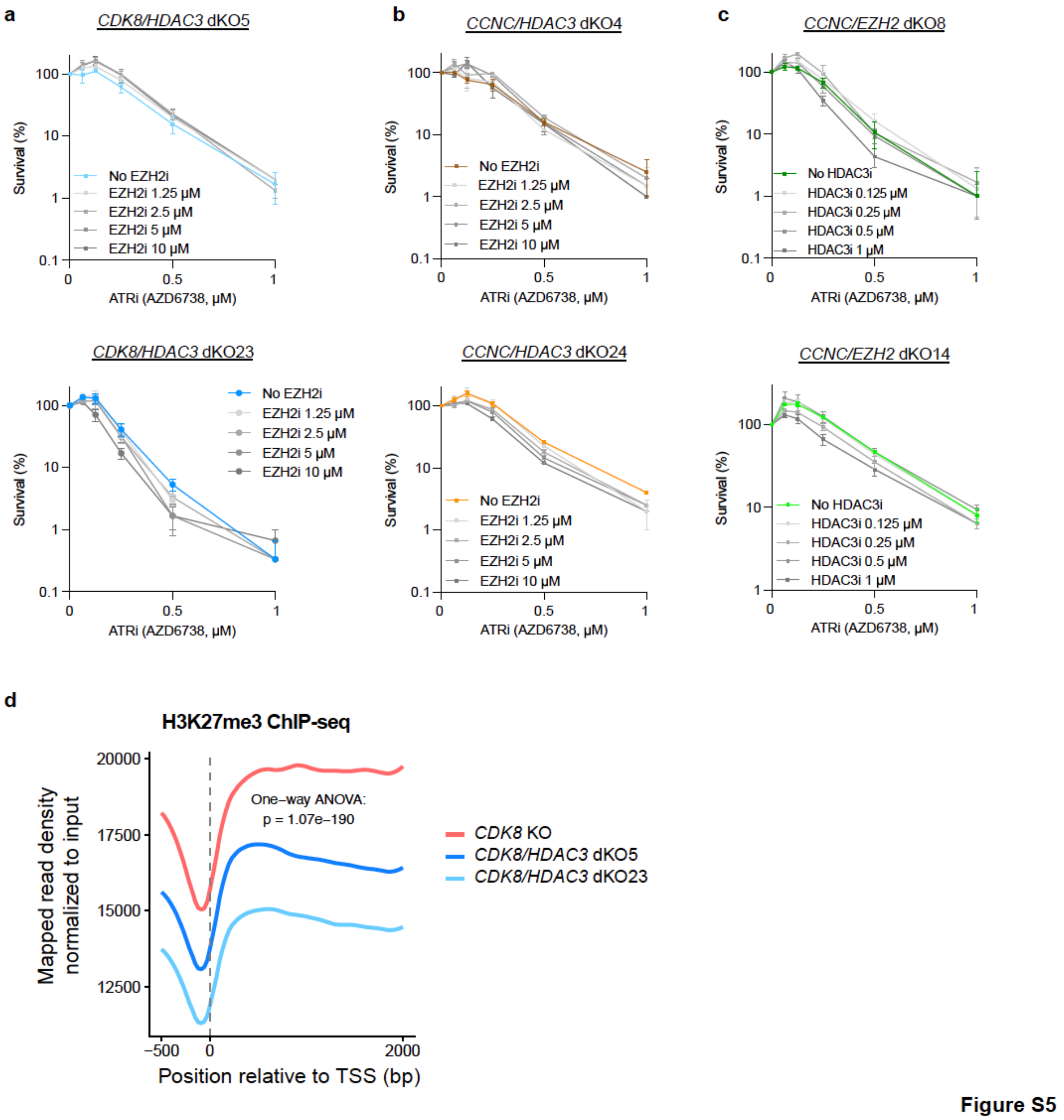

Supplementary Figure S5. HDAC3 and EZH2 operate in an epistatic manner. a. alarmBlue cell viability assay of CDK8/HDAC3 dKO clone #5 and clone #23 HAP1 cells upon treatment with ATRi or in combination with increasing concentrations of EZH2i. **b.** alarmBlue cell viability assay of CCNC/HDAC3 dKO clone #4 and clone #24 HAP1 cells upon treatment with ATRi or in combination with increasing concentrations of EZH2i. **c.** alarmBlue cell viability assay of the CCNC/EZH2 dKO clone #8 and clone #14 HAP1 cells upon treatment with ATRi or in combination with increasing concentrations of HDAC3i. In all panels, data represent means ± SEM from three biologically independent experiments. **d.** H3K27me3 ChIP-seq profile in WT, *CDK8* KO and *CDK8/HDAC3* dKO clones (#5, #23) (biological n=4). TSS, transcription start site.

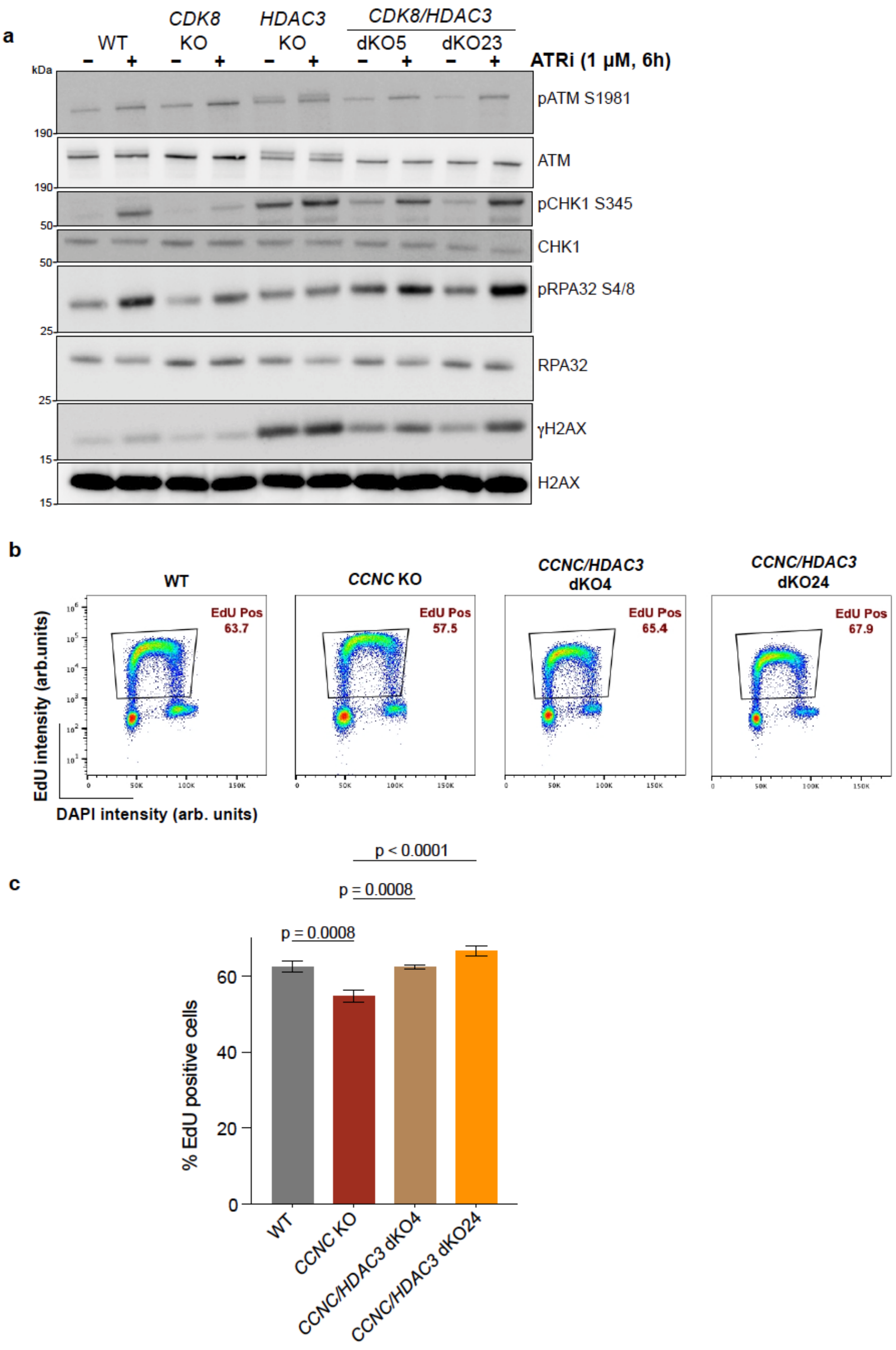

Figure S6

Supplementary Figure S6. HDAC3 loss induces DNA damage and replication stress in CDK8- and CCNC-deficient cells. a. Western blot analyses of replication stress and DSB formation markers in WT, *CDK8* KO, HDAC3 KO, and *CDK8/HDAC3* dKO (clones #5 and 23) HAP1 cells. Cells were treated with 1 µM ATRi for 6 h prior to lysis. Samples were run on separate gels: one gel for pATM, pCHK1, pRPA32, γH2AX, and another gel for ATM, CHK1, RPA32, H2AX. Data are representative of at least four independent experiments. **b.** EdU intensity versus DAPI plots for the indicated cell lines, with gated EdU-positive populations are shown. **c.** Quantification of EdU-positive populations from (b) in WT, *CCNC* KO, *CCNC/HDAC3* dKO4 and dKO24. Data represent mean ± SEM; biological n=8. Statistical analysis was performed using one-way ANOVA with multiple comparisons.

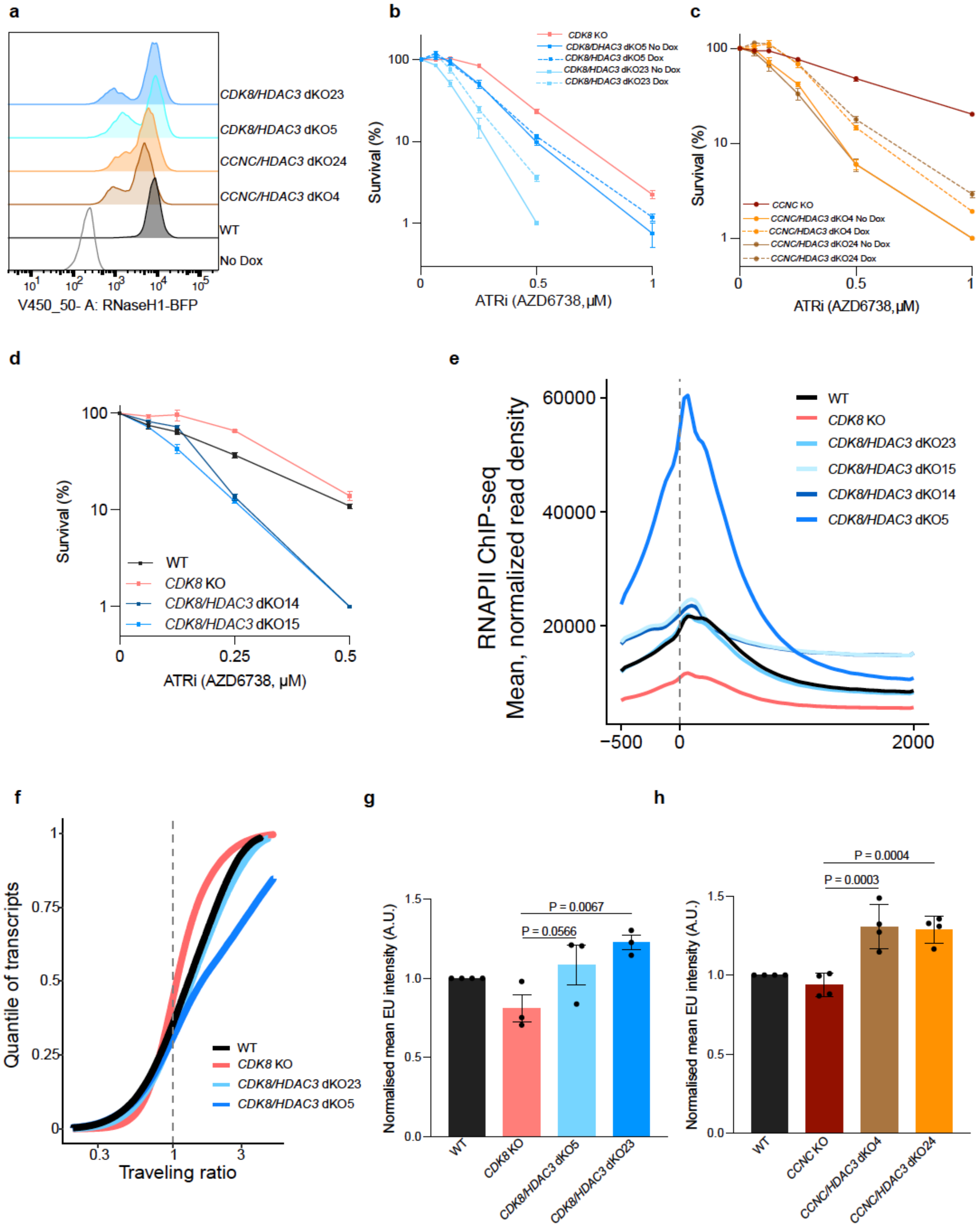

Figure S7

Supplementary Figure S7. HDAC3 loss induces transcriptional stress in *CDK8*- and *CCNC*-deficient cells. a. Flow cytometry analysis of BFP-tagged RNaseH1 expression in the indicated cell lines upon doxycycline (Dox) induction. A clear shift in the BFP-positive population was observed upon the addition of Dox compared to cells with no Dox, confirming successful RNaseH1 expression. **b, c.** alamarBlue cell viability assay of the indicated cell lines ± Dox induction of BFP-RNaseH1 expression, treated with ATRi. Data are shown as mean± SEM from four biological replicates. **d.** alamrBlue cell viability assay of additional CDK8/HDAC3 dKO clones (#14 and 15) treated with ATRi. Data are shown as mean± SEM

from three biological replicates. **e.** RNAPII ChIP-seq profile in WT, *CDK8* KO and *CDK8/HDAC3* dKO clones (#5, #23, #14 and #15) (biological n=4). TSS, transcription start site. **f.** RNAPII traveling ratio analysis for RNAPII transcripts in WT, *CDK8* KO, *CDK8/HDAC3* dKO #5 and #23, (biological n=4). Relative to WT, CDK8 depletion results in a shift towards lower values, which is reversed upon co-depleting HDAC3. **g, h.** Normalised mean EU intensities quantified by FACS in the indicated cell lines. Cells were incubated with 1 mM EU prior to sample collection. EU intensities for each repeat were normalised to WT cells. Error bars represent mean ± SEM (biological n=3 (g) and n=4 (f)).

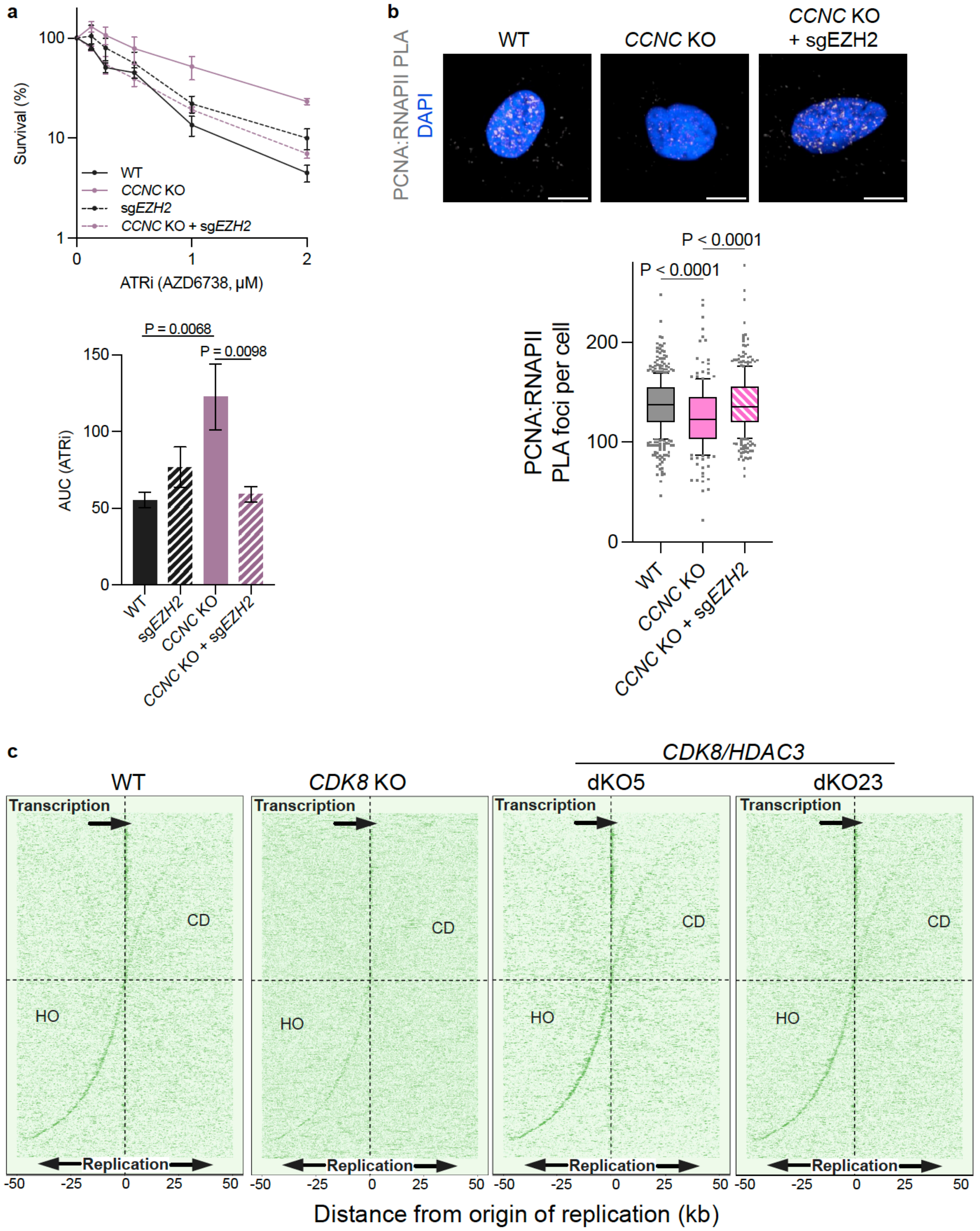

Figure S8

Supplementary Figure S8. HDAC3/EZH2 loss induces transcription-replication conflicts in *CDK8*- and *CCNC*-deficient cells. a. U2OS *CCNC*KO cells depleted of EZH2 (sg*EZH2*) display hypersensitivity to ATRi compared to WT U2OS cells. **Top-** alamarBlue cell viability assay of the indicated genotypes of U2OS cells treated with ATRi. Data are shown as mean ± SEM from three biological replicates. **Bottom-** Data represented as AUCs. Statistical analysis was performed using one-way ANOVA with multiple comparisons.

b. Top- representative Immunofluorescence images of PCNA:RNAPII PLA foci in WT, *CCNC* KO and *CCNC* KO infected with EZH2 sgRNA; nuclei were counterstained with DAPI. Scale bar= 10μm. **Bottom-** Quantification of PLA foci per cell from three independent biological replicates (n > 500 cells per replicate per group). Plots show median with 10–90th percentile range. Statistical analysis was performed using Two-way ANOVA test. **c.** Heat maps of RNAPII ChIP–seq in the indicated cell lines aligned around origins of replication. HO- head-on, CD- co-directional

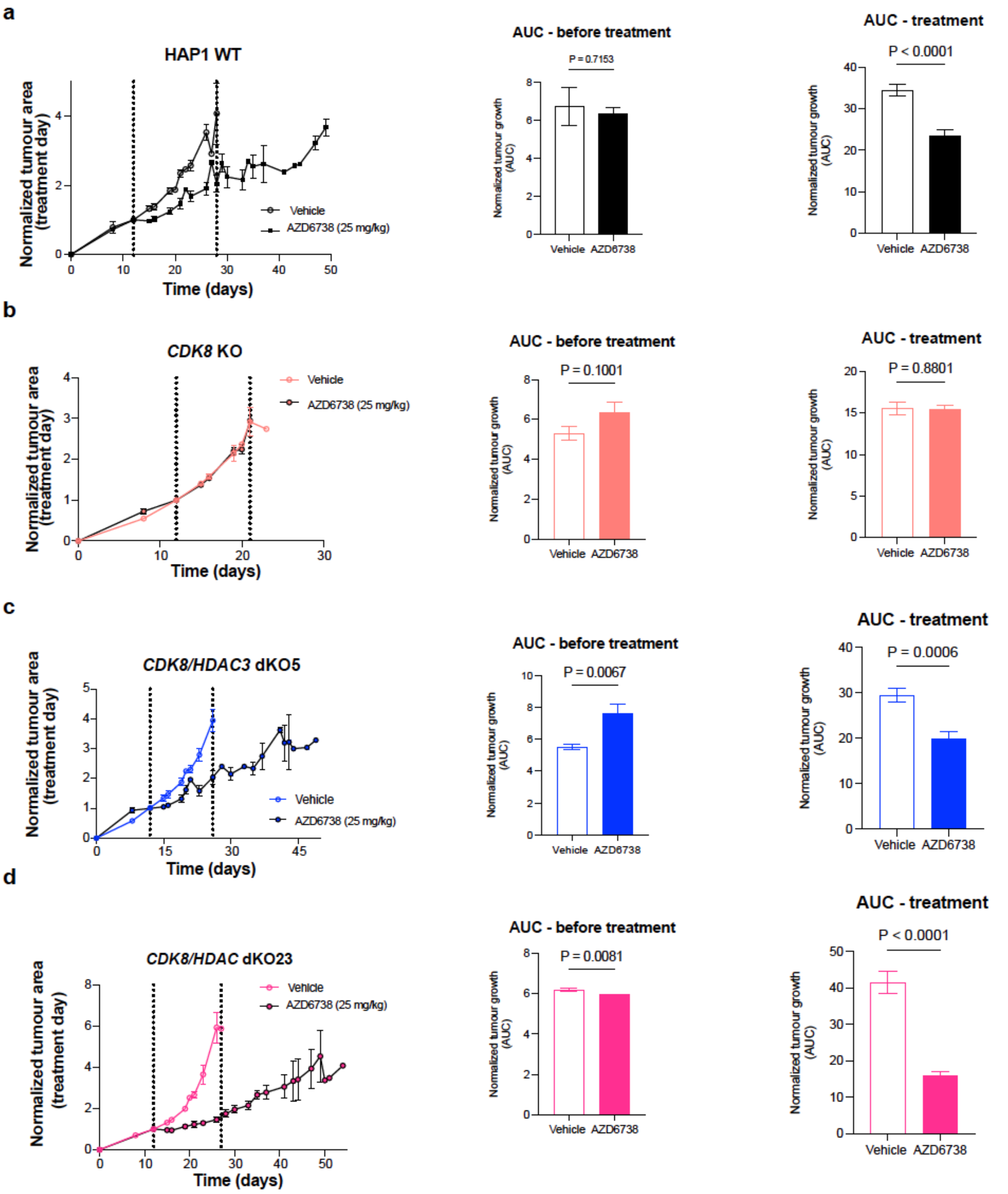

Figure S9

Supplementary Figure S9. HDAC3 loss sensitizes *CDK8*-deficient cells to ATRi *in vivo.* a-d. Tumour growth of xenografts derived from **a.** WT, **b.** *CDK8* KO, **c.** *CDK8/HDAC3* dKO5 and **d.** CDK8/HDAC3 dKO23 HAP1cells. Mice were treated daily with vehicle or AZD6738 by oral gavage. Tumour growth is shown as normalized tumour area (relative to day zero of treatment) ± SEM; n=8 mice per group. Right panels, AUC analysis of normalized tumour area before and during treatment. *P* values were determined using an unpaired two-tailed t-test.
